# Genetic interference of distinctive *Mycobacterium tuberculosis* peptidoglycan modifications enhances β-lactam susceptibility and reveals expression-sensitive host immune dynamics

**DOI:** 10.1038/s41598-026-59172-9

**Published:** 2026-06-23

**Authors:** Cátia Silveiro, Mariana Marques, Francisco Olivença, David Pires, Elsa Anes, Maria João Catalão

**Affiliations:** 1 Research Institute for Medicines (iMed.ULisboa), Faculty of Pharmacy, Universidade de Lisboa, 1649-003 Lisbon, Portugal; 2 Global Health and Tropical Medicine, GHTM, Instituto de Higiene E Medicina Tropical, IHMT, Universidade Nova de Lisboa, 1349-008 Lisbon, Portugal

**Keywords:** Tuberculosis, Antibiotic resistance, Peptidoglycan modifications, CRISPR interference, β-lactams, Diseases, Immunology, Microbiology, Molecular biology

## Abstract

The high mortality associated with tuberculosis (TB), alongside the lack of efficient therapeutics against emerging multidrug-resistant *Mycobacterium tuberculosis* (*Mtb*) strains, emphasizes the need for novel antitubercular targets. Mycobacterial peptidoglycan (PG), displaying characteristic modifications comprising the amidation of D-*iso*-glutamate (D-*i*Glu) and the *N*-glycolylation of muramic acid, is therefore a promising therapeutic target. The genes encoding the enzymes mediating these modifications (*murT*/*gatD* and *namH*) were silenced in *Mtb* using CRISPR interference (CRISPRi) to investigate their impact on β-lactam susceptibility and host immune responses. First, qRT-PCR confirmed successful target mRNA knockdown and phenotyping assays corroborated the essentiality of D-*i*Glu amidation for mycobacterial growth, in contrast to muramic acid *N*-glycolylation. The susceptibility assays demonstrated that both PG modifications promote β-lactam resistance. Indeed, we observed reductions in the minimum fractional inhibitory concentration index (FICI_min_) value for AMX/MEM + CLA and EMB combinations following the depletion of both PG modifications. Furthermore, D-*i*Glu amidation was found to promote *Mtb* fitness within THP-1-derived macrophages 6 days post-infection. Infection with MurT/GatD-depleted *Mtb* was associated with increased IL-1β and decreased IL-10, whereas NamH depletion was linked to increased IL-1β and IL-10 levels. Altogether, our findings unveiled the potential of targeting these PG modifications for the development of innovative therapeutic regimens against TB.

**Supplementary Information:**

The online version contains supplementary material available at https://doi.org/10.1038/s41598-026-59172-9.

## Introduction

Despite improvements in diagnostics and therapeutics, tuberculosis (TB) remains the deadliest infectious disease globally, causing over 1.25 million deaths and 10 million new cases annually^1^. The spread of multidrug-resistant strains of *Mycobacterium tuberculosis* (*Mtb*), coupled with limited additions to the anti-TB drug pipeline, has exacerbated disease burden^2^. The peculiar cell wall (CW) of *Mtb*, comprising a markedly modified peptidoglycan (PG), a profusely ramified arabinogalactan (AG), and long-chain mycolic acids (MA), is crucial for its antibiotic resistance and pathogenic success^3,4^. Since conventional antimycobacterials targets AG and MA biosynthesis, novel anti-TB agents should exploit the unique subtleties of mycobacterial PG (mPG).

Among these, a distinctive feature is the amidation of the α-carboxyl group of D-*iso*-glutamate (D-*i*Glu) in both lipid I/II, catalyzed by the MurT/GatD amidotransferase complex^3,4^. GatD hydrolyses L-glutamine into glutamate and ammonia, which is channelled to the active site of MurT and incorporated into D-*i*Glu in an ATP/Mg^2+^-dependent fashion, generating D-*iso*-glutamine (D-*i*Gln)^5–7^. The essential *murT/gatD* operon is conserved among *Mtb* clinical isolates^7–9^, and inhibition of D-*i*Glu amidation has been associated with impaired growth and increased β-lactam and lysozyme susceptibility in *Staphylococcus aureus* and *Mycobacterium smegmatis* (*Msm*)^8–11^. Additionally, D-*i*Gln-containing lipid II is the favoured substrate for PG polymerization in *Mtb*^9,12,13^ and may facilitate evasion of NOD1/2-mediated immune recognition^14,15^. Accordingly, mice vaccinated with a MurT/GatD-depleted *Mycobacterium bovis* Bacillus Calmette–Guérin (*M. bovis* BCG) strain exhibited heightened pro-inflammatory responses and superior protection versus BCG^16^.

Another hallmark mPG modification is the *N*-glycolylation of muramic acid, catalyzed by *N*-acetyl muramic acid hydroxylase (NamH), which hydroxylates the *N*-acetyl moiety to generate *N*-glycolyl^17^. Consequently, mPG incorporates both *N*-acetyl and *N*-glycolyl muramic acid derivatives, interspersed with *N*-acetylglucosamine residues ^17^. Although *namH* is non-essential for growth^18,19^, its conservation among *Mtb* clinical strains suggests a role in mycobacterial fitness^8,20^. Notably, NamH depletion increases β-lactam and lysozyme susceptibility^8,17^. Whereas some studies state that *N*-glycolylated PG stimulates NOD2-mediated immunity^21,22^, others suggest it may reduce recognition^15^.

Additional characteristic mPG features include the amidation of *meso*-diaminopimelic acid (*m*-DAP) on lipid II mediated by the AsnB amidotransferase and a predominance of non-classical PG cross-links catalyzed by L,D-transpeptidases (Ldts)^3,23,24^, resistant to conventional β-lactams^25^. This, along with the impermeability of the MA layer, and the expression of a chromosomally encoded β-lactamase BlaC^26^, has hindered the clinical use of β-lactams against *Mtb*^27^. Nonetheless, carbapenem/β-lactamase inhibitor combinations^28^ have shown efficacy against multidrug-resistant tuberculosis (MDR-TB)^29–32^. Given the WHO’s call for further evidence on β-lactam efficacy in TB therapy, distinct β-lactam susceptibility patterns in *Mtb* have been reported^33,34^.

Recently, we demonstrated that D-*i*Glu amidation and muramic acid *N*-glycolylation modulate β-lactam resistance in *Msm* and contribute to its intracellular survival^8^. To investigate the applicability of these findings in the pathogen, the CRISPRi platform^35^ was implemented to target these PG modifications in *Mtb*, revealing species-specific differences in essentiality, intracellular fitness, and immune modulation.

## Methods

### Bacterial strains, plasmids, culture conditions, cell lines, and antibiotics

The list of bacterial strains, plasmids, and cell lines used in this work are detailed in Supplementary Table S1. To amplify the CRISPRi backbone PLJR965 (Addgene ID: 115163) and for cloning, *Escherichia coli* (*E. coli*) strains were grown at 37 °C with shaking in Luria–Bertani (LB) medium (Merck). *Mtb* strains were grown in Middlebrook 7H9 broth (BD™ Biosciences) supplemented with 0.2% glycerol (ThermoScientific), 10% OADC (oleic acid; albumin; dextrose; catalase), and 0.05% tyloxapol (Sigma-Aldrich) or in 7H10 agar (BD™ Biosciences) plates supplemented with 0.5% glycerol and 10% OADC at 37 °C, without shaking. The manipulation of *Mtb* strains was performed in a BSL-3 laboratory. When appropriate, 25 μg/mL kanamycin (NZYTech) were added to the media for plasmid selection. Target mRNA knockdown in mycobacterial cultures was induced by adding 100 ng*/*mL anhydrotetracycline (ATc) (Sigma-Aldrich) daily. To cultivate THP-1 cells, RPMI 1640 medium (Gibco) was enriched with 10% fetal bovine serum (Gibco), 1% L-Glutamine (Gibco), 1% HEPES (Gibco), 1% sodium pyruvate (Gibco), and 1% non-essential amino acids (Gibco). The cultures were maintained at 37 °C in the presence of 5% CO_2_. Stocks of amoxicillin (AMX), cefotaxime (CTX), ethambutol (EMB), isoniazid (INH) and meropenem (MEM) (Sigma-Aldrich) were dissolved in sterile MilliQ water at a final concentration of 1.28 mg/mL^8^. The ATc stocks were prepared at 10 mg/mL in dimethyl sulfoxide (DMSO) cell culture grade (AppliChem)^8^. Potassium clavulanate (CLA) (Sigma-Aldrich) stocks were dissolved in phosphate buffer at 0.1 M, pH 6.0^8^.

### Construction of the knockdown mutants using CRISPRi

Cloning protocols were performed as previously detailed^8^. To attain efficient knockdown, the coding strand or the promoter region of the genes of interest (GOIs)—*murT* (*Rv3712*), *gatD* (*Rv3713*), and *namH* (*Rv3818*)—were targeted. The strength of the chosen protospacer adjacent motif (PAM) is frequently correlated with the extent of knockdown efficiency and the ensuing growth inhibition phenotypes^35,36^. According to the predicted essentiality of the GOIs, their template strands were screened for PAM sequences^35^. Strong PAMs were employed for the repression of putatively non-essential genes whereas medium-to-low strength PAMs mediated the knockdown of presumably essential genes. Besides accounting for gene essentiality, sgRNA design (Supplementary Table S2) was optimized by combining distinct PAM strengths and target sites to achieve differential silencing profiles.

The CRISPRi backbone PLJR965 was amplified in *E. coli* JM109, digested, and purified as formerly explained^8^. To clone the targeting sgRNAs, two complementary primers were designed (Supplementary Table S3) and synthesized (Eurofins Genomics) with blunt ends to reconstitute the sgRNA promoter and the dCas9 handle sequences. This was accomplished following primer annealing and ligation of the annealed oligos to the backbone vector at 22 °C for 30 min, facilitated by T4 DNA ligase (400 U/µL; NEB).

To obtain the intended gene-targeting CRISPRi plasmids, chemically competent cells of *E. coli* were transformed with the ligation mixture by double heat-shock. Using the NZYMiniprep kit (NZYTech), recombinant DNA was isolated from the resultant transformants and then confirmed by Sanger sequencing to match the desired constructs. To generate the knockdown mutants (KDMs) in *Mtb*, electrocompetent cells were transformed with 300 ng of recombinant DNA in the presence of 10% glycerol, as previously reported^8,37^.

### Mycobacterial RNA isolation

To collect bacterial cultures for total RNA isolation, *Mtb* strains were grown to the exponential phase, normalized to a theoretical optical density at 600 nm (OD_600_) of 0.1–0.2, and divided into equal volumes devoid of or containing 100 ng/mL ATc^36^. After an incubation period of 72 h at 37 °C, approximately 6 × 10^8^ cells were harvested by centrifugation at 3000 × *g* for 5–10 min at 4 °C ^36^, ressuspended and incubated in 500 μL of RNAprotect Bacteria Reagent (Qiagen) for 1 h, and stored at −80 °C. When necessary, the cells were thawed and pelleted by centrifugation at 5000 × *g* for 5 min. To induce lysis, 350 µL of buffer NR (NZYTech) and 3.5 µL of β-mercaptoethanol (Merck) were added to the cells, which were then fully disrupted using BeadBug™ (Benchmark Scientific). Bead-beating was carried out for 4 cycles at maximum speed, each lasting 1 min, interleaved with 1-min incubations on ice. After a spin-down, the lysate was loaded into the NZYSpin Homogenization column (NZYTech), and the workflow proceeded in conformity with the instructions provided by the NZYTotal RNA Isolation Kit. To elute RNA, the column was incubated with 20–30 μL of DEPC water (Invitrogen) at 60 °C for 10 min, followed by centrifugation and a second incubation-centrifugation step to maximize yield. A rigorous treatment with TurboDNase (Invitrogen) was then conducted to prevent contamination with genomic DNA. Nanodrop™ was used to appraise the concentration and purity ratios of the eluted RNA and the efficacy of TurboDNase treatment was assessed by PCR amplification of the constitutive gene *sigA* (*Rv2703*), using NZYTaq DNA polymerase (NZYTech).

### Assessment of target gene repression by quantitative reverse-transcription PCR (qRT-PCR)

To confirm efficient repression of target genes, 100 ng of purified RNA were reverse-transcribed into cDNA^8,38^ using the NZY First-Strand cDNA Synthesis Kit (NZYTech). To quantify cDNA, qPCR reactions were prepared using the NZYSupreme qPCR Green Master Mix (2 ×), ROX (NZYTech) and performed on a QuantStudio™ 7 Flex Real-Time PCR System (Applied Biosystems). The qPCR program comprised an initial 2 min polymerase activation step at 95 °C, followed by 40 cycles of 5 s denaturation at 95 °C and 30 s annealing/extension at 60 °C. Primers were designed using Primer 3 (https://primer3.ut.ee/) to produce a specific 100–200 bps amplicon (Supplementary Table S4), and amplification efficiency was confirmed to be within the 80–110% range by standard curve analysis. Two technical replicates and a negative control were included in each run, and the average C_T_ was determined. Data analysis was performed using the Comparative C_T_ method, with *Mtb* WT serving as calibrator and *sigA* as the internal control gene^35,36,39^. The quantification of target mRNA sequences was performed using three biological replicates.

### Phenotyping assays

To ascertain the importance of distinctive mPG modifications to bacterial growth, phenotypic characterization was performed using spotting dilution and growth curve assays.

Spotting dilution assays were conducted as previously described^8^. Briefly, *Mtb* cultures were grown to the exponential phase, adjusted to a theoretical OD_600_ of 0.001, and subjected to 1:2 dilution in a 96-well plate. Then, 5 μL of each bacterial suspension were plated onto 7H10 + OADC (+ ATc) plates at 37 °C for approximately 15 days. More than three independent experiments were completed to ensure reproducibility.

To monitor bacterial growth, *Mtb* liquid cultures were grown to the exponential phase, normalized to a theoretical OD_600_ of 0.005–0.01 and, further incubated at 37 °C with and without inducer for 264 h. The concentration of ATc was replenished to 100 ng/mL every 24 h. The OD_600_ was measured at 96, 120, 168, 192, and 264 h across three independent experiments. Growth was evaluated by plotting the mean OD_600_ values versus the incubation time for each strain under study.

### Minimum inhibitory concentration (MIC) determination

To unveil changes in antibiotic susceptibility upon target gene repression, the minimum inhibitory concentrations of three β-lactams (amoxicillin, cefotaxime, and meropenem), in the presence and absence of the β-lactamase inhibitor clavulanate (CLA), and of two first-line antimycobacterials (INH and EMB) were ascertained. Succinctly, the compounds under assessment were diluted to their predefined initial concentrations (512 μg/mL for β-lactams and 128 μg/mL for antitubercular agents) and subjected to a 1:2 serial dilution in supplemented 7H9 media. CLA was used at a final concentration of 2.5 μg/mL. In the last two rows, positive (bacteria only) and negative (media only) control wells were included. The cultures of *Mtb* were first diluted to a theoretical OD_600_ of 0.1 and split into two equal volumes, one of which was subjected to a pre-depletion period of 3 days with 100 ng/mL ATc^40^. Following this period, the cultures were normalized to a OD_600_ of 0.002, induced with a final concentration of 100 ng/mL ATc, and dispensed into the designated wells to a final concentration of 10^5^ CFU/mL. The plates were then incubated at 37 °C for 10–12 days. The MIC was annotated as the minimum concentration of antibiotic that completely suppressed observable bacterial growth. Three independent experiments were carried out for each strain, and the median MIC values were calculated.

### Disk diffusion assays

To assess potential changes in antibiotic susceptibility on solid media upon induced target interference, disk diffusion assays were performed with AMX + CLA, CTX + CLA, and MEM + CLA, adapting previously published protocols^26^. Briefly, *Mtb* cultures were grown to the exponential phase, diluted to a theoretical OD_600_ of 0.1, and spread onto the 7H10 + OADC (+ ATc) plates, which were then incubated at 37 °C for 1–2 days to create a mycobacterial lawn. Afterwards, β-lactams were diluted to the required concentrations for susceptibility testing (AMX: 512 µg/mL; CTX: 512 µg/mL; MEM: 128 µg/mL; CLA: 5 µg/mL), and 20 μL of each compound were transferred to the respective 9 mm filter paper disks (Filterlab), which were allowed to dry and placed onto the lawn. Following 10–13 days of incubation at 37 °C, the diameter of the zone of inhibition (halo) was measured using a ruler. The results depict the mean diameter of the zone of inhibition (in cm) from a minimum of three biological replicates.

### Checkerboard assays

To evaluate the interactions between frontline agents (INH or EMB), β-lactams, and the CRISPRi-mediated interference of target genes (simulating the prospective chemical inhibition of NamH and MurT/GatD), checkerboard assays were performed. Since previous observations showed a synergistic effect between EMB and amoxicillin-clavulanate (AMX + CLA) or meropenem-clavulanate (MEM + CLA) in *Mtb* H37Rv^41^, we selected these combinations for further antibiotic interaction assessment. The minimum fractional inhibitory concentration indices (FICIs) were calculated to measure the extent of these interactions and whether these were classified as synergy (FICI ≤ 0.5), additive effect (0.5 < FICI ≤ 1), indifference (1 < FICI ≤ 4), or antagonism (FICI > 4)^42^.

The checkerboard assays were conducted as previously described^41^, with few modifications. Two or three days before the assay, *Mtb* cultures were diluted to a theoretical OD_600_ of 0.1 and split into two equal volumes, one of which was subjected to a pre-depletion period of 2–3 days with 100 ng/mL ATc^40^. On the day of the assay, the antibiotics were prepared at four times (dilution factor) the initial test concentration (corresponding to the quadruple of the respective individual MIC) and serially diluted 1:2 in supplemented 7H9. Using a multichannel pipette, 50 µL of each antibiotic under study was transferred to the wells of a 96-well plate, along the abscissa or ordinate, thus forming an 8 × 8 matrix with a total volume of 100 µL. To preserve a humidified atmosphere, water or medium was added to the remaining wells. The cultures were then centrifuged at 3000 × *g* for 5 min, resuspended in 1 mL of supplemented 7H9 media, submitted to an ultrasonic bath for 5 min, and centrifuged at 500 × *g* for 15 s to obtain a clump-free suspension. After, the cultures were diluted to a theoretical OD_600_ of 0.002 in supplemented 7H9 media, to which CLA (5 μg/mL) and ATc (100 ng/mL) were added at the indicated final concentration, when appropriate. After, the matrix wells were inoculated with 100 µL of each bacterial suspension, producing a final concentration of 10^5^ CFU/mL. The OD₆₀₀ of the wells was measured using an Infinite M200 Pro microplate reader (TECAN) following 10–12 days of incubation at 37 °C.

In this case, the MIC was annotated as the concentration achieving ≥ 99% inhibition of mycobacterial growth relative to the positive control. The fractional inhibitory concentration (FIC) of each tested antibiotic was determined by dividing the MIC of the antibiotic when used in combination by its individual MIC (e.g., FIC_EMB_ = MIC_EMBxAMX+CLA_/MIC_EMB_). The FIC index (FICI) is obtained by adding the individual FICs of the antibiotics being tested in each well (e.g., FICI for interaction assessment between EMB and AMX + CLA = MIC_EMB×AMX+CLA/_MIC_EMB_ + MIC_EMB×AMX+CLA_/MIC_AMX+CLA_). The presented results show the FICI values as the mean ± SEM of at least three independent experiments.

### THP-1 infection assays

To investigate the role of the distinctive modifications of mPG in the intracellular fitness of *Mtb*, THP-1-derived macrophages were infected with the control and mutant strains. THP-1 monocytes were routinely passaged every 3–4 days to maintain a density of 2 × 10^5^ cells/mL. As needed, the cells were seeded at a density of 1.5 × 10^5^ cells/well in 48-well flat-bottom tissue culture plates and treated with 20 nM phorbol 12-myristate 13-acetate (PMA; Sigma-Aldrich) for 72 h to induce differentiation into resting macrophages^41,43,44^. On the day of THP-1 seeding, *Mtb* cultures were diluted to an OD_600_ of approximately 0.05 and split into uninduced and induced conditions, which were treated with 100 ng/mL ATc^40^. The cultures were then incubated at 37 °C for 72 h, after which the concentration of inducer was replenished.

One day prior to infection, the cell media containing PMA was exchanged by prewarmed supplemented PMA-deprived RPMI media. For the infection, log phase *Mtb* cultures were harvested by centrifugation at 3,000 × *g* for 5 min, washed with 5 mL of phosphate-buffered saline (PBS) 1 × (Gibco), and resuspended in supplemented RPMI media^41,43^. The bacterial suspension was then submitted to an ultrasonic bath for 5 min, and the remaining clumps were pelleted by centrifugation at 500 × *g* for 15 s, enabling the recovery of a single-cell suspension^43,45^. Following the removal of the cell media from the plates, THP-1-derived macrophages were infected with the mycobacterial suspensions at a multiplicity of infection (MOI) of 0.25 and, incubated at 37 °C with 5% CO_2_ for various timepoints (1 d; 3 d; 6 d), consistent with a long-term infection model that minimizes macrophage cell death^46,47^. At this point, induced cultures were added 100 ng/mL ATc. Following an internalization period of 3 h, the macrophages were washed twice with PBS 1 × to remove extracellular bacteria and cultivated in prewarmed supplemented RPMI media^41,43^, devoid of or containing 250 ng/mL ATc, necessary to sustain long-term target knockdown^40^. Prewarmed supplemented RPMI media and ATc (225 ng/mL) were replenished 3 days after infection onset (T_3_) to sustain both cell growth and interference^40^.

### Intracellular CFU evaluation

After the specified incubation times (T_1_, T_3_, and T_6_), the medium was discarded, and macrophages were lysed with 0.05% IGEPAL (Merck) at 37 °C for 15 min^41,45^. Subsequently, the lysates were serially diluted in sterile water and plated onto 7H10 + OADC plates, which underwent incubation at 37 °C with 5% CO_2_ for 2–3 weeks until colonies were visible^41,45^. Finally, the colony-forming units (CFU) from three independent biological replicates, each comprising three technical replicates, were counted using an Olympus CK2 Inverted Phase Contrast Microscope.

### Immune response assessment of infected THP-1-derived macrophages via qRT-PCR

To ascertain whether the amidation of D-*i*Glu or the *N*-glycolylation of muramic acid induce differential immune responses upon infection with *Mtb*, THP-1-derived macrophages were infected with each of the study strains. After 6 days of infection, THP-1 cells were lysed and transcript levels of pro-inflammatory cytokines tumor necrosis factor alpha (TNF-α) and interleukin-1 β (IL-1β), and of the anti-inflammatory cytokine interleukin-10 (IL-10), were measured by qRT-PCR. For these assays, THP-1 monocytes were seeded at a density of 5 × 10^5^ cells/well in 12-well flat-bottom tissue culture plates and differentiated into resting macrophages via the addition of 20 nM PMA for 72 h^41,43^. The preparation of bacterial cultures and macrophage infection (MOI of 0.25) proceeded as aforementioned. The lysis of infected macrophages was promoted by vigorous cell resuspension in 394 µL of buffer NR (NZYTech) and 4 µL of β-mercaptoethanol (Merck). The obtained lysates were transferred to an NZYSpin Homogenization column (NZYTech), and the ensuing steps were performed following the instructions of the NZYTotal RNA Isolation Kit^45^. The isolation of purified RNA was facilitated by a two-step elution with 20–30 μL of 60 °C DEPC-treated water, and 100 ng of total RNA were used as template for cDNA synthesis using the NZY First-Strand cDNA Synthesis Kit (NZYTech)^45^. The qRT-PCR assay was carried out as described above, with the following primers (Supplementary Table S5). Each run included two technical replicates and a negative control, and the average C_T_ value was calculated. Data analysis was carried out using the Comparative C_T_ method, with non-infected macrophages as the calibrator sample and *GAPDH* (*glyceraldehyde 3-phosphate dehydrogenase*) as the endogenous control gene^43,45^. The mRNA levels of TNF-α, IL-1β, and IL-10 were quantified using three biological replicates.

### Statistical analysis

All statistical analyses were carried out using GraphPad Prism (version 8.4.3.686), and results are expressed as mean ± SEM, unless mentioned otherwise. The Shapiro–Wilk test was employed in normality testing. The ordinary one-way ANOVA was used to compare multiple groups, followed by pairwise analyses of preselected groups using the Holm-Sidak test. All the prerequisites for the employed tests were verified.

## Results

### Construction of CRISPRi knockdown mutants

**Fig. 1:**
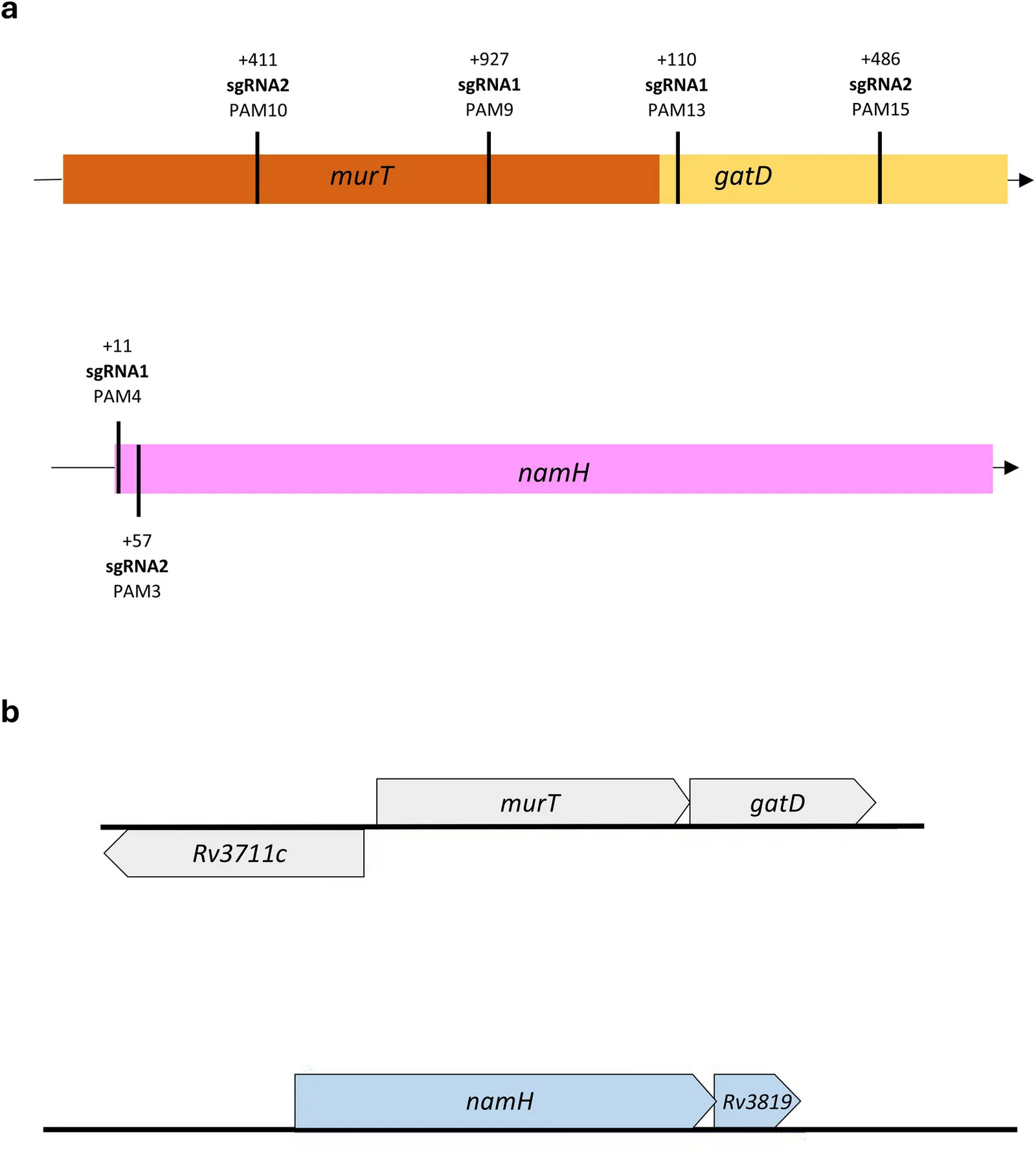
(**a**) Distribution of sgRNA targeting sequences along the *murT*/*gatD* operon and the *namH* gene in *Mtb*. Different sites were targeted along the genes to assess the differential repression efficiency of CRISPRi. (**b**) The gene local context of the target genes *murT*, *gatD*, and *namH* should be considered due to potential downstream/upstream polar effects. The *murT* and *gatD* genes are co-transcribed under the regulation of a single promoter. The *Rv3711c* gene precedes *murT* but is transcribed in the opposite direction. A single promoter guides the conjoint transcription of the *namH* and *Rv3819* genes by RNA polymerase.

To engineer the knockdown mutants of our GOIs in *Mtb*, the ATc-regulated dCas9_Sth1_-mediated CRISPRi system was employed^35^. Distinct PAM sequences were employed to assess differential knockdown efficiency owing to PAM strength and/or target site^8^ (Fig. 1a). To prevent lethal phenotypes, PAMs were selected based on predicted GOI essentiality^8^. Since our GOIs are operonic, local gene context was also considered to deter polar effects^35,38,39^ (Fig. 1b). Interference plasmids were verified by Sanger Sequencing (Supplementary Fig. S1).

**Fig. 2:**
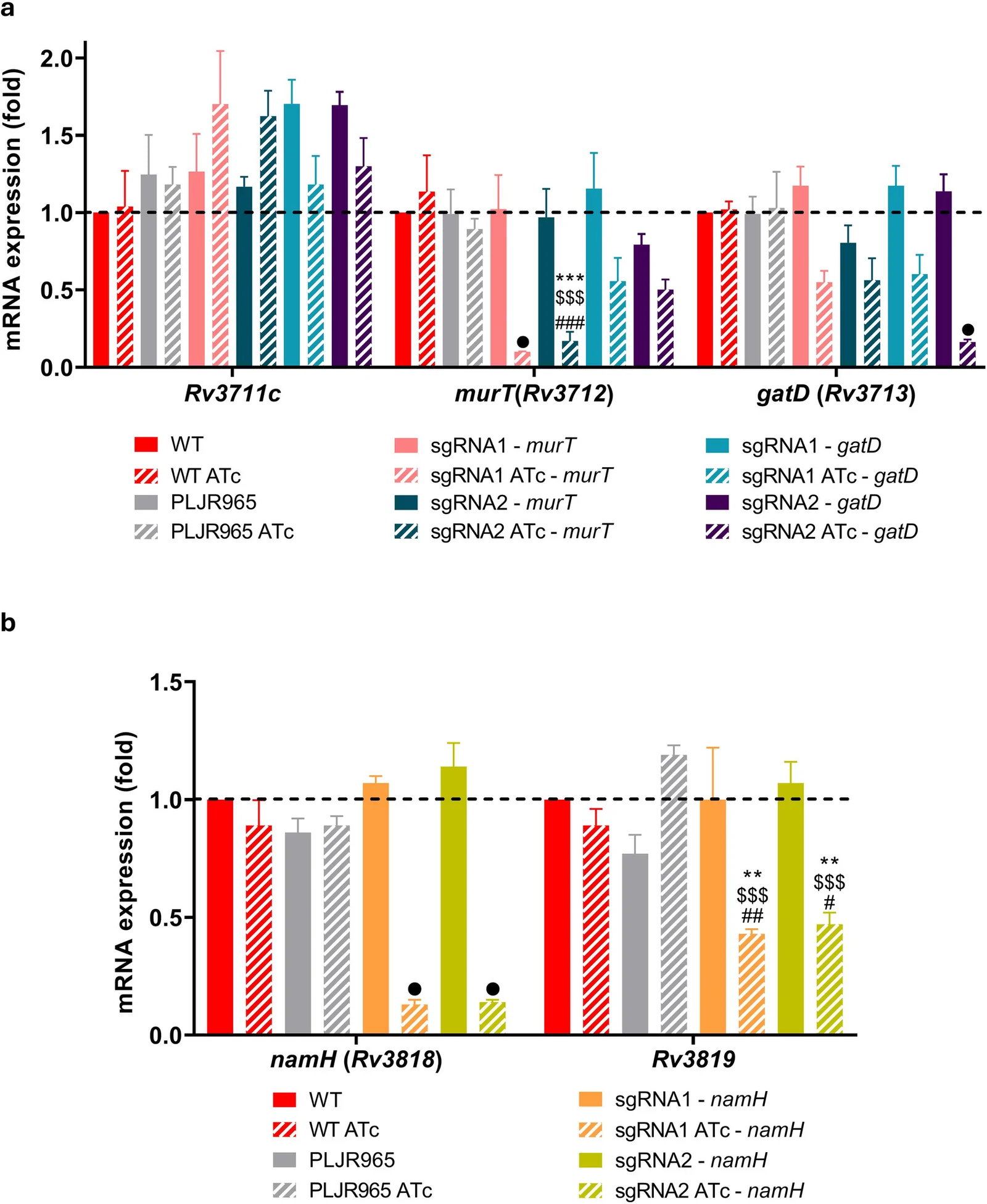
Mean relative mRNA expression levels of target genes, normalized to *sigA*, at 72 h post-induction, with (stripped bars) and without (smooth bars) 100 ng/mL ATc (n = 3): (**a**) *murT* and *gatD* knockdown; (**b**) *namH* knockdown. The expression levels of the adjacent genes (**a**) *Rv3711c*, the gene immediately upstream of *murT*, and (**b**) *Rv3819*, the gene downstream of *namH*, were also evaluated to check for polar effects. The dashed lines show the WT sample as calibrator. Error bars show the standard error of the mean (SEM). Multiple comparisons were made using one-way ANOVA: * *P* < 0.05; ** *P* < 0.01; *** *P* < 0.001. Significant differences are indicated with symbols: # compared to *Mtb* WT ATc, $ compared to *Mtb* PLJR965 ATc, * compared the respective uninduced control. The • symbol indicates a highly significant difference (*P* < 0.0001) from all controls.

The normalized mRNA expression of *murT*, *gatD, namH,* and adjacent genes was determined in the control strains *Mtb* WT and PLJR965, and in the KDMs, with and without inducer, at 72 h post-induction (Fig. 2a, b).

The results demonstrate that *murT* was successfully silenced in all induced KDMs (Fig. 2a). The sgRNA1-directed silencing of *murT* caused a highly significant reduction in transcript levels (*P* < 0.0001) relative to WT ATc (11.3-fold), PLJR965 ATc (8.9-fold), and the respective uninduced control (10.1-fold). Likewise, sgRNA2-directed *murT* knockdown caused a very significant repression versus WT ATc (6.7-fold; *P* = 0.0002), PLJR965 ATc (5.2-fold; *P* = 0.0005), and the uninduced control (5.7-fold; *P* = 0.0005). Differences in PAM strength and sgRNA length directly accounted for greater *murT* repression with sgRNA1-*murT* (PAM9, + 927 bp) compared to sgRNA2-*murT* (PAM10, + 411 bp). Conversely, only one of the targeting sgRNAs efficiently repressed *gatD* (Fig. 2a). Indeed, sgRNA2-mediated *gatD* knockdown caused a highly significant decrease in its expression (*P* < 0.0001) versus WT ATc (6.2-fold), PLJR965 ATc (6.3-fold), and the respective uninduced control (7.0-fold). Upon CRISPRi-mediated silencing of the *murT*/*gatD* operon, no significant polar effects were observed in the adjacent gene *Rv3711c*, which encodes the DNA polymerase III ε subunit (DnaQ) (Fig. 2a).

Additionally, a highly significant knockdown of *namH* (*P* < 0.0001) was attained with both sgRNA1-*namH* and sgRNA2-*namH* compared to WT ATc (6.9-fold vs. 6.4-fold), PLJR965 ATc (6.9-fold vs. 6.4-fold), and the respective uninduced mutant (8.3-fold) (Fig. 2b). The repression efficiency of sgRNA1-*namH* (PAM4, + 11 bp) and sgRNA2-*namH* (PAM3, + 57 bp) was comparable, as repression differences between strong PAMs are negligible^35^. Moreover, both sgRNAs target the 5’ region of *namH*: sgRNA1-*namH* targets the promoter, while sgRNA2-*namH* targets the first nucleotides of the ORF. Both sgRNAs caused significant downstream polar effects on *Rv3819* (Fig. 2b), which are likely to partially affect the ensuing phenotypes. While non-essential^18,19,48^ and functionally uncharacterized, *Rv3819* encodes a protein containing a PS00012 phosphopantetheine attachment site.

### Phenotyping reveals the essentiality of* murT/gatD* and non-essentiality of* namH*

**Fig. 3:**
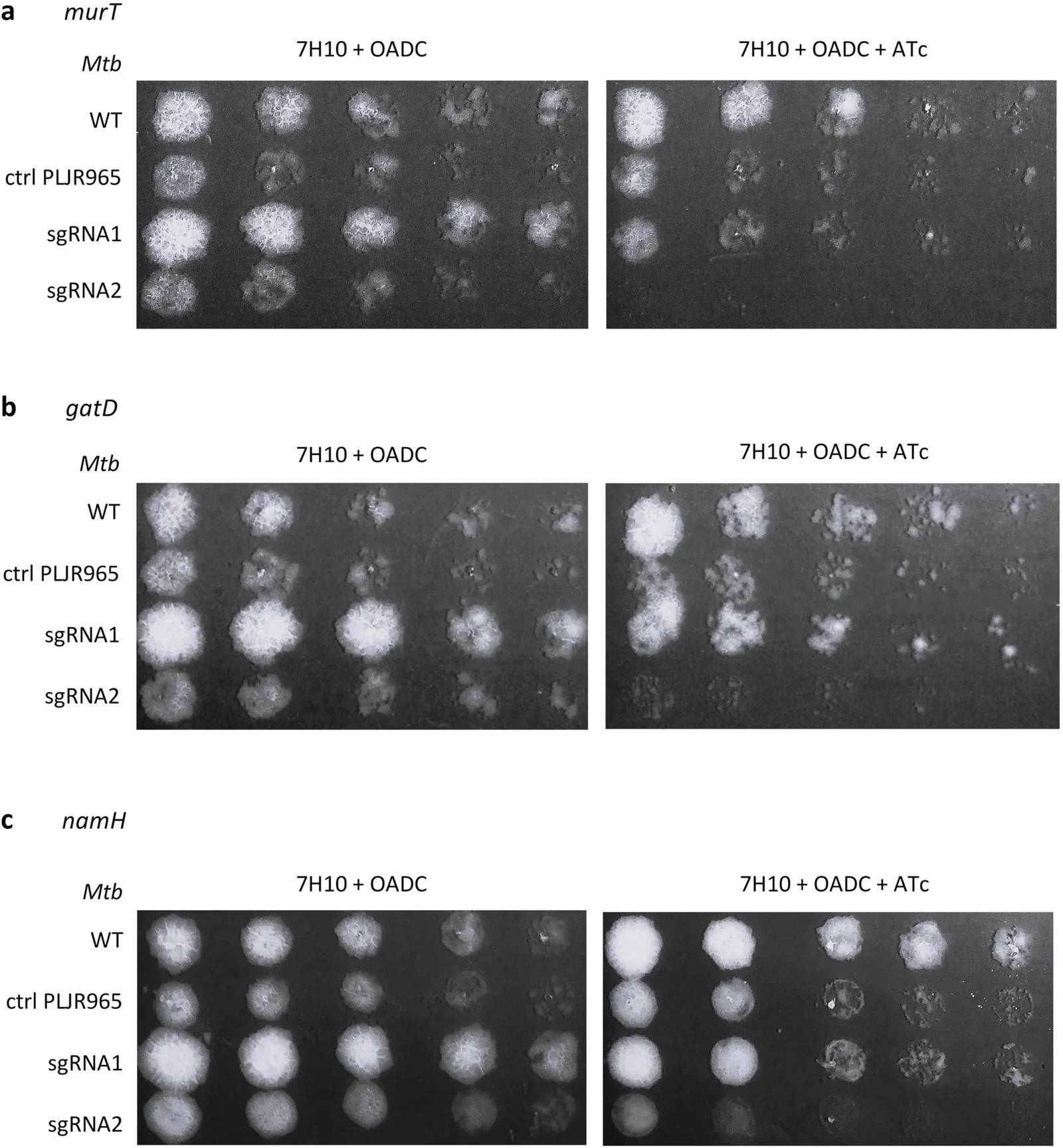
Spotting dilution assays of the *M. tuberculosis* knockdown mutants (n = 3): (**a**) *murT* knockdown mutants; (**b**) *gatD* knockdown mutants; (**c**) *namH* knockdown mutants. Negative controls (*Mtb* WT and PLJR965) were included in all experiments. Each culture was first normalized to an OD_600_ of 0.001, then serially diluted twofold, and spotted onto agar plates.

**Fig. 4:**
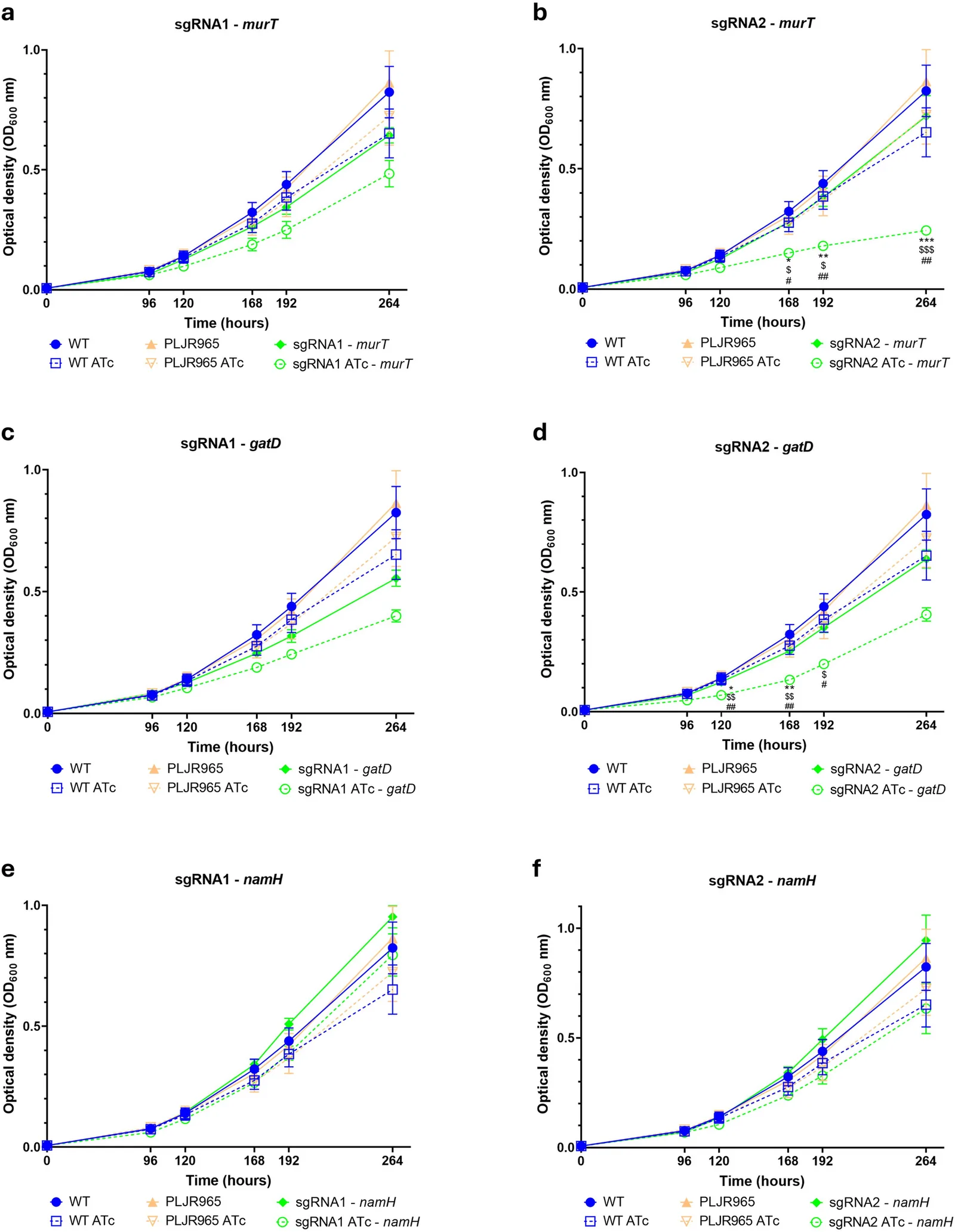
Growth curves of the *M. tuberculosis* knockdown mutants, in the absence or presence of 100 ng/mL ATc (n = 3): (**a**, **b**) *murT* knockdown mutants; (**c**, **d**) *gatD* knockdown mutants; (**e**, **f**) *namH* knockdown mutants. Negative controls (*Mtb* WT and PLJR965) were included in all experiments. Error bars show the standard error of the mean (SEM). Multiple comparisons were made using one-way ANOVA, with significance levels: * *P* < 0.05; ** *P* < 0.01; *** *P* < 0.001. Significant differences are indicated with symbols: # compared to *Mtb* WT ATc, $ compared to *Mtb* PLJR965 ATc, * compared to the respective uninduced control.

To phenotypically characterize the KDMs, spotting dilution and growth curve assays were conducted (Figs. 3 and 4). The spotting dilution assays demonstrated that the growth of the control strain *Mtb* WT was not impacted by inducer addition. In contrast, *Mtb* PLJR965 displayed only a slight growth reduction on solid media containing ATc, suggesting that the expression of exogenous dCas9_Sth1_ may be somewhat toxic to *Mtb*. The results confirmed the essentiality of the *murT*/*gatD* operon for *Mtb* survival, as its knockdown caused severe growth impairment, particularly when mediated by potent sgRNAs. Regarding *murT* knockdown (*murT*^-^), sgRNA2-mediated silencing (PAM10, + 411 bp) completely abrogated *Mtb* growth, while sgRNA1-*murT* (PAM9, + 927 bp) provoked only a slight growth defect (Fig. 3a). Although the employed sgRNAs theoretically mediate comparable knockdown levels, sgRNA2-*murT* guides dCas9_Sth1_ binding to the 5’-region of *murT*, which may cause RNA polymerase dissociation at an early stage of transcription, thus generating a small mRNA likely to be degraded. On the other hand, sgRNA1-*murT* targets a site near the 3’-region of *murT*, with dCas9_Sth1_-mediated steric blockage of the RNA polymerase leading to the production of a larger mRNA, likely to produce a semi-functional MurT protein. Regarding *gatD* knockdown (*gatD*^*-*^), sgRNA1-*gatD* (PAM13, + 110 bp) caused a minor growth reduction whereas sgRNA2-*gatD* (PAM15, + 486 bp) severely impacted bacterial growth, despite employing a theoretically “weaker” PAM (Fig. 3b, Supplementary Table S2). These phenotypes reflect the greater knockdown mediated by sgRNA2-*gatD* compared to sgRNA1-*gatD*. Moreover, sgRNA1-mediated *namH* repression did not cause any major growth changes, whereas sgRNA2-mediated *namH* knockdown provoked an observable growth deficit (Fig. 3c). These observations suggest that NamH activity impacts *Mtb* growth in these conditions. The domain organization of NamH is shown in Supplementary Fig. S2.

The growth curves showed that *murT* repression severely affected growth in liquid media, in line with the spotting assays (Fig. 3a). sgRNA2-mediated silencing (PAM10, + 411 bp) led to a severe reduction in *Mtb* growth, whereas sgRNA1-*murT* (PAM9, + 927 bp) caused only a modest growth defect (Fig. 4a, b).

Regarding *gatD* repression, sgRNA2-mediated knockdown caused a more substantial growth impairment (T_120_, T_168_, and T_192_ h) than sgRNA1-*gatD* (Fig. 4c, d). This is consistent with the higher knockdown efficiency of sgRNA2-*gatD* (Fig. 2a) and with the spotting assays (Fig. 3b). As for *namH* repression, sgRNA1-*namH* did not cause any observable changes in *Mtb* growth while sgRNA2-*namH* prompted a subtle growth reduction (Fig. 4e, f), in accordance with the spotting assays (Fig. 3c).

### The activity of MurT/GatD and NamH promotes β-lactam resistance

**Fig. 5:**
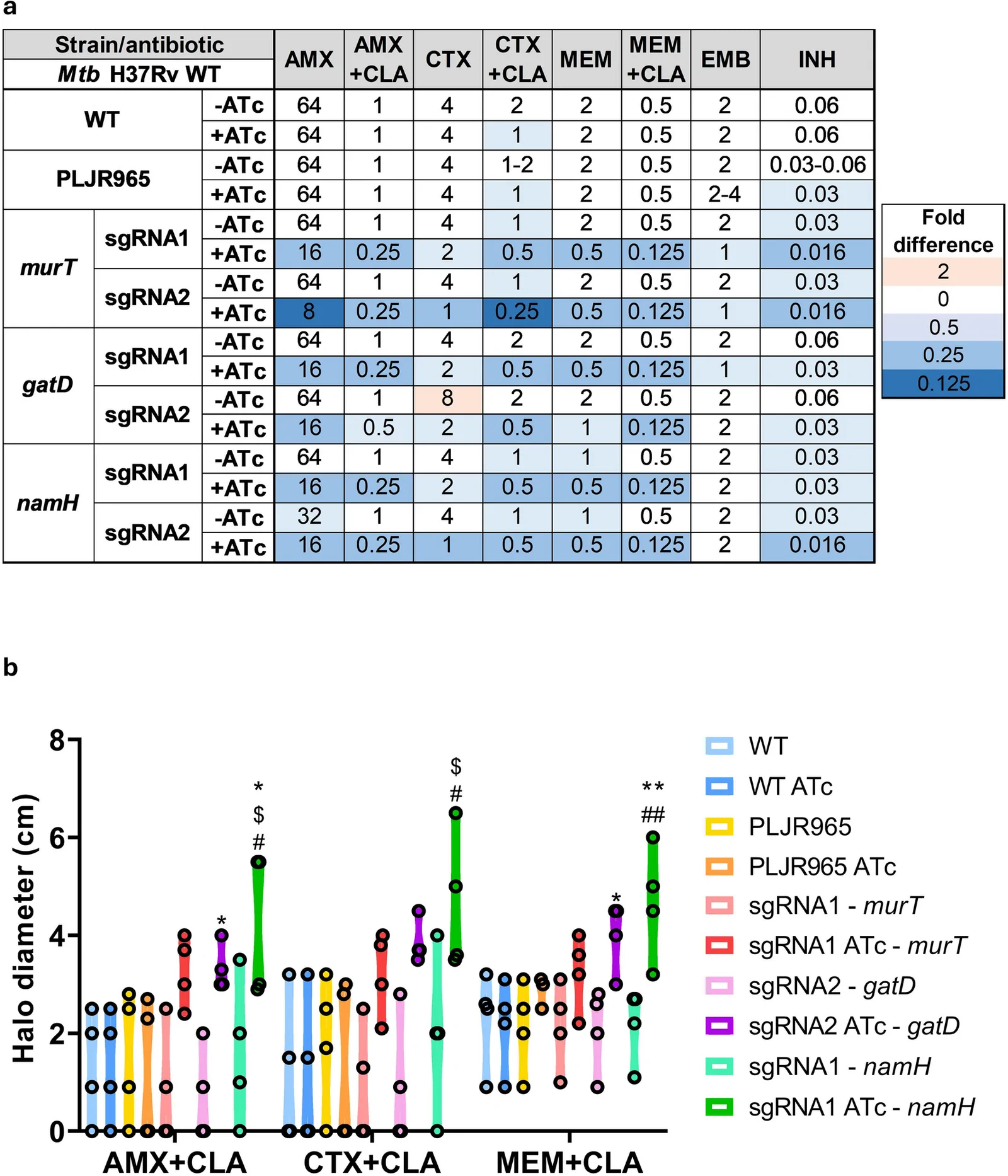
(**a**) Heatmap of the fold differences in the minimum inhibitory concentrations (MICs) of the knockdown mutants constructed in *M. tuberculosis*, with and without 100 ng/mL of ATc. Columns represent the antibiotics and rows represent the strains. The median MIC (in µg/mL) of each antibiotic for each strain is shown in the respective intersection, and colours depict an increase (red shades) or decrease (blue shades) in the value when compared to the MICs of *Mtb* WT. AMX, amoxicillin; CLA, clavulanate; CTX, cefotaxime; EMB, ethambutol; INH, isoniazid; MEM, meropenem. (**b**) Violin plot of the diameter of the zone of inhibition (in cm) for each combination of beta-lactam/CLA with the indicated knockdown mutants constructed in *Mtb* WT for the *murT* (sgRNA1), *gatD* (sgRNA2) and *namH* (sgRNA1) genes with and without 100 ng/mL ATc (n ≥ 3). AMX, amoxicillin; CLA, clavulanate; CTX, cefotaxime; MEM, meropenem. Violin plots were clipped at y = 0, as negative halo diameters are not biologically feasible and open black dots represent individual data points. Multiple comparisons were made using one-way ANOVA, with significance levels: * *P* < 0.05; ** *P* < 0.01; *** *P* < 0.001. Significant differences are indicated with symbols: # compared to *Mtb* WT ATc, $ compared to *Mtb* PLJR965 ATc, * compared to the respective uninduced control.

To determine how characteristic mPG modifications influence the antibiotic susceptibility profile of *Mtb*, the MICs of EMB, INH, and three β-lactams, in the absence or presence of CLA, were determined against control strains and KDMs (Fig. 5a). As predicted, ATc induction did not considerably affect MIC values for the control strains (*Mtb* H37Rv WT, *Mtb*::PLJR965). Conversely, all KDMs displayed increased β-lactam susceptibility, irrespective of the targeted gene.

Generally, median MICs for the KDMs revealed that depletion of D-*i*Glu amidation (*murT*/*gatD*^-^) and of the *N*-glycolylation of muramic acid (*namH*^-^) promoted increased susceptibility to β-lactams. Regarding *murT* and *namH* repression, the observed susceptibility phenotypes correlated well with the growth deficits displayed by the respective KDMs in the spotting assays (Fig. 3a, c). Despite sgRNA1-*murT* being more efficient at silencing *murT* (Fig. 2a), sgRNA2-mediated *murT* knockdown resulted in a lethal phenotype in the spotting assays (Fig. 3a) and heightened β-lactam susceptibility (Fig. 5a). Similarly, sgRNA2-mediated *namH* silencing led to reduced growth in the spotting assays (Fig. 3c) and was also associated with β-lactam hypersusceptibility (Fig. 5a). Surprisingly, sgRNA1-mediated *gatD* silencing did not result in a significant knockdown (Fig. 2b) but caused greater β-lactam susceptibility differences than sgRNA2-mediated *gatD* knockdown (Fig. 5a). It seems that a minimal repression mediated by a sgRNA that targets the 5’-region of *gatD* has a greater impact on β-lactam susceptibility than significant repression mediated by a sgRNA targeting a region near the 3’-end of *gatD*. Overall, sgRNA2-mediated *murT* silencing caused the largest MIC differences compared to *Mtb* WT, with AMX and CTX + CLA MICs decreasing by eightfold (Fig. 5a).

For AMX, all induced KDMs displayed substantial susceptibility differences (≥ fourfold) relative to WT. Likewise, considerable differences in AMX + CLA susceptibility were observed in all KDMs except for the induced *Mtb*::PLJR965-sgRNA2-*gatD* KDM. The MICs of CTX remained mostly constant, except for the induced *Mtb*::PLJR965-sgRNA2-*murT* and *Mtb*::PLJR965-sgRNA2-*namH* KDMs, which exhibited a striking increase in β-lactam susceptibility. Regarding CTX + CLA, all induced KDMs showed considerable susceptibility differences (≥ fourfold) relative to WT. Regarding MEM, substantial susceptibility differences (≥ fourfold) were found in all KDMs except for the induced *Mtb*::PLJR965-sgRNA2-*gatD* KDM. In the case of MEM + CLA, all induced KDMs presented considerable susceptibility differences versus WT. The addition of CLA to β-lactams provoked a notable 64-fold reduction in AMX MICs, while the MICs of CTX and MEM were only reduced by fourfold. The MIC of MEM + CLA against the induced KDMs was 0.125 μg/mL, being fourfold lower than the WT MIC. Altogether, MEM + CLA seems to synergize well with MurT/GatD and NamH inhibition. Among the tested β-lactams, MEM consistently exhibited the lowest MICs, being the most effective at inhibiting *Mtb* growth. Finally, the MICs of EMB remained stable, while three induced KDMs displayed increased INH susceptibility, most pronounced in the *murT* KDMs.

### Disk diffusion assays demonstrate that MurT/GatD and NamH modulate β-lactam resistance

To validate these findings, disk diffusion assays were performed with control strains and KDMs to assess susceptibility differences to β-lactams combined with CLA (AMX + CLA, CTX + CLA, MEM + CLA) on solid media following CRISPRi induction (Fig. 5b).

Based on the qRT-PCR results, only one sgRNA per target gene was selected for further assays, using two criteria: i) efficient silencing of the respective target gene; ii) sufficient growth of the resulting KDMs in the phenotyping assays to ensure that the observed susceptibility and immune response differences arise from gene knockdown rather than lethal effects, which is particularly important for essential genes. The chosen sgRNAs were sgRNA1 for the *namH* and *murT* genes and sgRNA2 for the *gatD* gene.

To evaluate susceptibility differences on solid media, antibiotics were prepared at concentrations producing distinguishable zones of inhibition. For all β-lactams, these concentrations were 256 or 512-fold higher than the MICs determined in liquid media. As anticipated, susceptibility of WT and PLJR965 to β-lactams remained relatively stable, even with inducer (Fig. 5b). Again, depletion of characteristic mPG modifications increased β-lactam susceptibility (Fig. 5b). Regarding AMX + CLA, sgRNA2-mediated *gatD* repression resulted in significantly heightened susceptibility versus the corresponding uninduced control (*P* = 0.0457). Additionally, a significant increase in susceptibility was observed upon sgRNA1-mediated *namH* silencing relative to WT ATc (*P* = 0.0196), PLJR965 ATc (*P* = 0.0142), and the uninduced control (*P* = 0.0457). For CTX + CLA, susceptibility was significantly increased upon *namH* silencing versus WT ATc (*P* = 0.0118) and PLJR965 ATc (*P* = 0.0253). Susceptibility to MEM + CLA was also significantly increased following sgRNA2-mediated *gatD* repression compared to the respective uninduced control (*P* = 0.0407). Likewise, sgRNA1-mediated *namH* knockdown led to pronounced increase in MEM + CLA susceptibility versus WT ATc (*P* = 0.0033) and the uninduced control (*P* = 0.0033). Generally, NamH depletion caused significant β-lactam hypersusceptibility. Furthermore, MEM + CLA was the most potent β-lactam against *Mtb* growth, as indicated by a broader zone of inhibition compared to AMX + CLA and CTX + CLA (Fig. 5b).

### NamH depletion synergizes with EMB and AMX/MEM + CLA

**Fig. 6:**
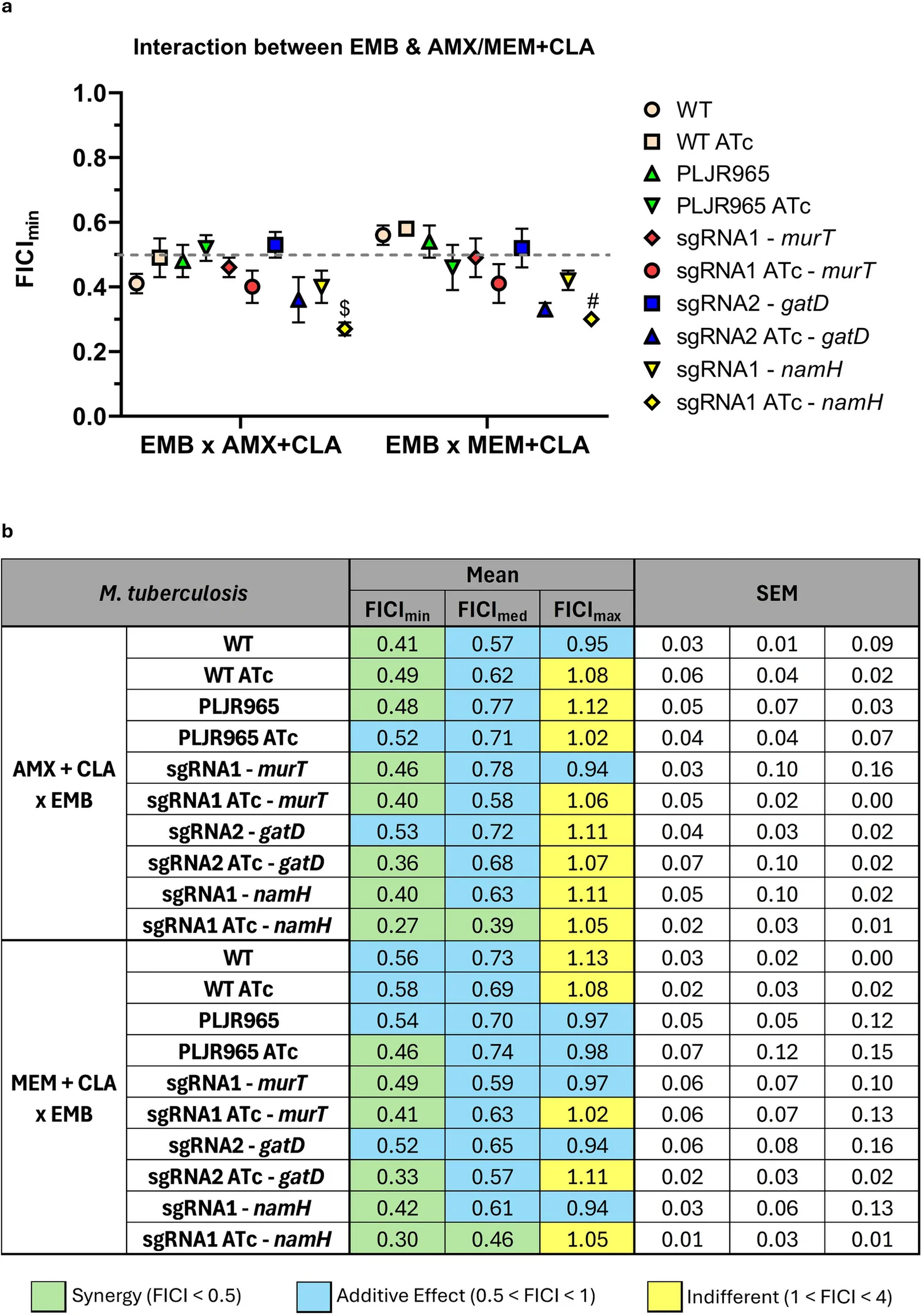
(**a**) Representation of the minimum fractional inhibition indices (FICI_min_) assessing the interaction between EMB and the beta-lactams AMX + CLA and MEM + CLA were determined for the control strains (*Mtb* WT and PLJR965) and the *murT* (sgRNA1), *gatD* (sgRNA2), and *namH* (sgRNA1) knockdown mutants, with and without 100 ng/mL ATc (n ≥ 3). The dotted grey line represents the FICI_min_ value underneath which interactions are considered synergistic (FICI_min_ ≤ 0.5). Multiple comparisons were made using one-way ANOVA: * *P* < 0.05. Significant differences are indicated with symbols: # compared to *Mtb* WT ATc and $ compared to *Mtb* PLJR965 ATc. (**b**) Heatmap of the detailed outputs of the checkerboard assays assessing the interaction between EMB and the beta-lactams AMX + CLA and MEM + CLA for the control strains (*Mtb* WT and PLJR965) and the *murT* (sgRNA1), *gatD* (sgRNA2) and *namH* (sgRNA1) knockdown mutants, with and without 100 ng/mL ATc (n ≥ 3). The results show the values of minimum fractional inhibitory concentration (FIC) index (FICI_min_), the median FICI (FICI_med_), and the maximum FICI (FICI_max_) calculated as the average of at least three independent replicates for each combination and each strain. The color of the cells depicts the type of interaction according to the FICI value. The concentration of CLA was fixed at 5 µg/mL. AMX, amoxicillin; CLA, clavulanate; EMB, ethambutol; MEM, meropenem. Error bars show the standard error of the mean (SEM).

To evaluate interactions between frontline agents, β-lactams, and CRISPRi-mediated target inhibition (simulating the prospective chemical inhibition of mPG modifications), checkerboard assays were performed with control strains and selected KDMs (Fig. 6a, b). Given preceding evidence of synergistic effects between EMB and AMX + CLA or MEM + CLA in *Mtb* (48), these combinations were further assessed. Fractional inhibitory concentration indices (FICIs) were calculated and interactions between EMB and AMX/MEM + CLA were classified as synergistic, additive, indifferent, or antagonistic.

Checkerboard assays showed that target repression led to reduced minimum FICI (FICI_min_) values for the interaction between EMB and AMX/MEM + CLA (Fig. 6a, b). All strains, except for *Mtb*::PLJR965 ATc and the uninduced *Mtb*::PLJR965-sgRNA2-*gatD*, displayed synergy between EMB and AMX + CLA. Synergies were less common with EMB plus MEM + CLA, where some interactions were classified as additive. Notably, NamH depletion significantly decreased the FICI_min_ between EMB plus AMX + CLA (*P* = 0.0205) or MEM + CLA (*P* = 0.0362) versus PLJR965 ATc and WT ATc, respectively (Fig. 6a).

### D-*i*Glu amidation contributes to intracellular fitness

To investigate how distinctive mPG modifications modulate host–pathogen interactions, THP-1-derived macrophages were infected with control strains and KDMs at a MOI of 0.25, and intracellular bacterial burden (CFU/mL, encompassing the combined effects of bacterial uptake, survival, and replication) was evaluated at 1, 3, and 6 days post-infection.

**Fig. 7:**
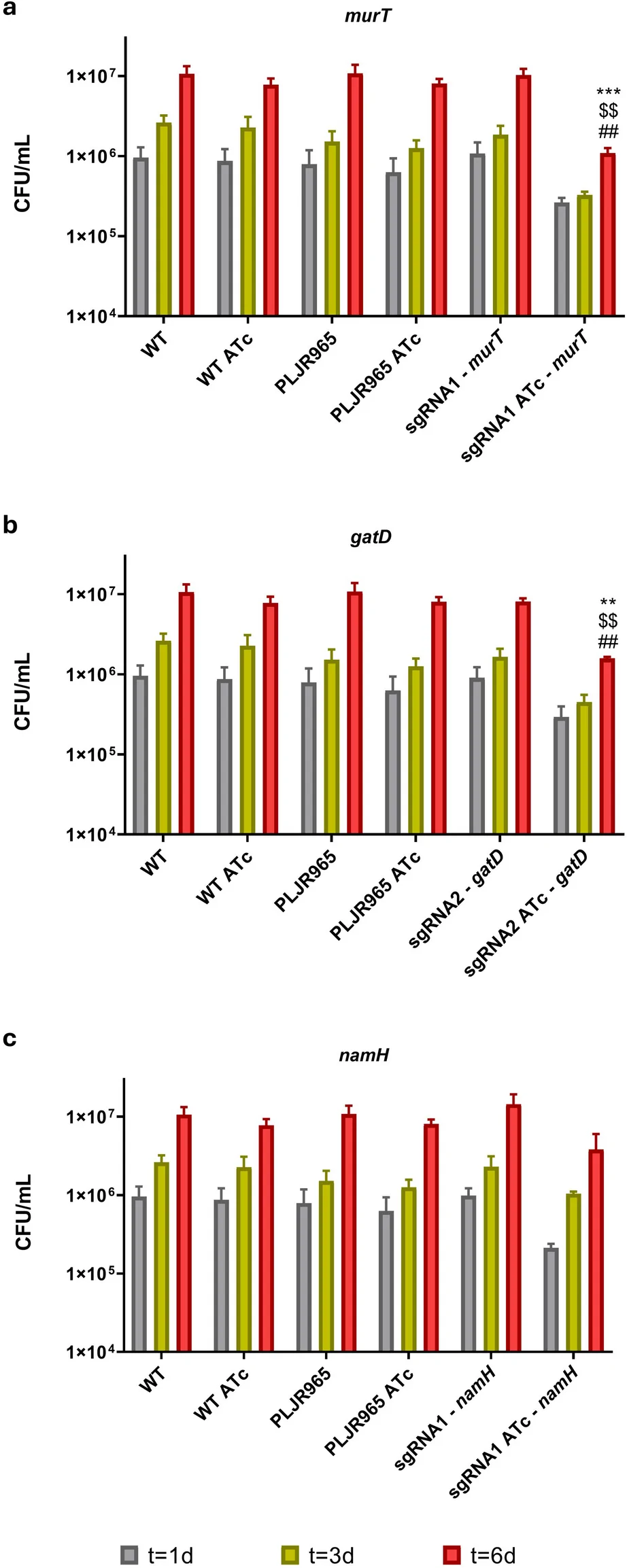
Logarithmic representation of the mean bacterial fitness (in CFU/mL) after disruption of THP-1-derived macrophages infected with the control strains (*Mtb* WT and PLJR965) and with the knockdown mutants, with and without ATc (n = 3): (**a**) *murT* knockdown mutants (sgRNA1); (**b**) *gatD* knockdown mutants (sgRNA2); (**c**) *namH* knockdown mutants (sgRNA1). Error bars show the standard error of the mean (SEM). Multiple comparisons were made using one-way ANOVA: * *P* < 0.05; ** *P* < 0.01; *** *P* < 0.001. Significant differences are indicated with symbols: # compared to *Mtb* WT ATc, $ compared to *Mtb* PLJR965 ATc, * compared to the respective uninduced control.

As anticipated, the intracellular CFU of *Mtb* increased over time, and the addition of inducer had little effect on *Mtb* WT and PLJR965 survival within THP-1 macrophages (Fig. 7a–c). Notably, CRISPRi-mediated *murT*/*gatD* knockdown reduced *Mtb* intracellular CFU compared to controls, with significant differences at 6 days post-infection. Indeed, sgRNA1-mediated *murT* knockdown prompted a highly significant decline in intracellular bacterial load at T_6_ versus WT ATc (*P* = 0.0015), PLJR965 ATc (*P* = 0.0012), and the respective uninduced control (*P* = 0.0005) (Fig. 7a). Similarly, sgRNA2-mediated *gatD* knockdown led to a very significant intracellular CFU drop at T_6_ relative to WT ATc (*P* = 0.0054), PLJR965 ATc (*P* = 0.0039), and the respective uninduced control (*P* = 0.0027) (Fig. 7b). Conversely, the reduction in intracellular *Mtb* burden caused by sgRNA1-mediated *namH* knockdown was not statistically significant (Fig. 7c).

Normalization to the PLJR965 ATc control demonstrated that *murT*/*gatD* knockdown produced a substantial and progressive *Mtb* fitness reduction, while *namH* knockdown caused a milder decrease (Supplementary Fig. S3).

### D-*i*Glu amidation is a mechanism of immune evasion

To uncover whether these PG modifications influence the immune response, THP-1-derived macrophages were lysed 6 days post-infection, and mRNA levels of the pro-inflammatory cytokines TNF-α and IL-1β, and anti-inflammatory cytokine IL-10 were evaluated by qRT-PCR.

**Fig. 8:**
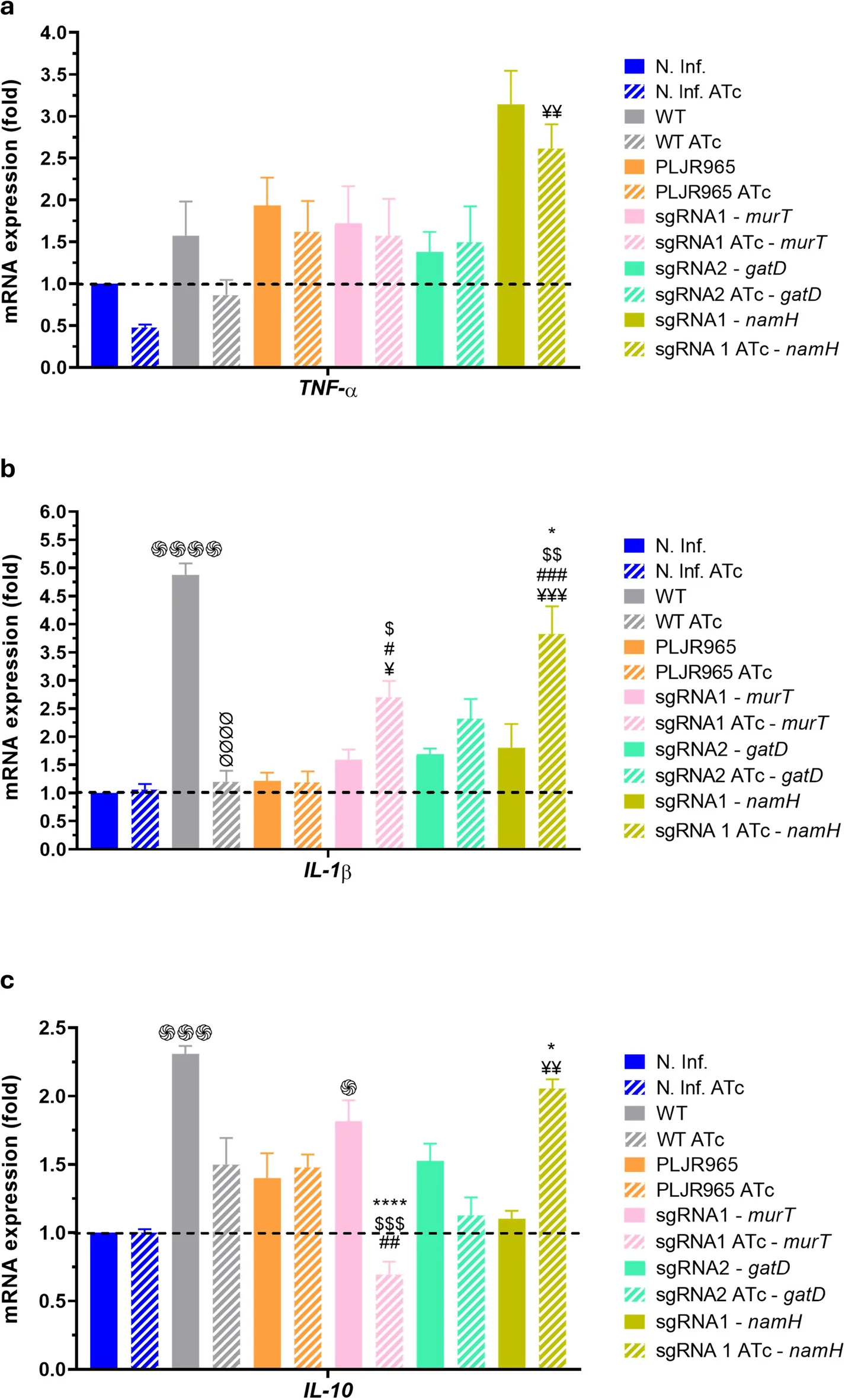
mRNA expression levels of (**a**) TNF-α, (**b**) IL-1β*,* (**c**) IL-10 assessed in THP-1-derived macrophages infected with the controls (*Mtb* WT and PLJR965) and the *murT*, *gatD*, and *namH* knockdown mutants, with and without ATc (n = 3). Cytokine expression was assessed at 6 days post-infection and normalized to *GAPDH*. Smooth bars represent infections with uninduced strains, while striped bars depict ATc-induced conditions. Dashed lines represent non-infected macrophages as the calibrator. Error bars show the standard error of the mean (SEM). Multiple comparisons were made using one-way ANOVA: * *P* < 0.05; ** *P* < 0.01; *** *P* < 0.001. Significant differences are depicted with symbols: ֍ compared to non-infected macrophages, ¥ compared to N. Inf. ATc, Ø compared to *Mtb* WT, # compared to *Mtb* WT ATc, $ compared to *Mtb* PLJR965 ATc, * compared to the respective uninduced control.

Overall, TNF-α levels showed no major changes across conditions, with slightly higher expression in infected versus non-infected macrophages (N. Inf.) (Fig. 8a). Non-infected ATc-treated macrophages (N. Inf. ATc) displayed about half the TNF-α expression of their untreated controls, although without significance. Remarkably, TNF-α levels were significantly increased upon infection with the induced *Mtb*::PLJR965-sgRNA1-*namH* KDM, compared to N. Inf. ATc (*P* = 0.0066) (Fig. 8a). However, no significant differences were observed between induced and non-induced sgRNA1-*namH* strains (Fig. 8a).

Furthermore, non-infected untreated and ATc-treated macrophages exhibited similar IL-1β levels. Nevertheless, infection with *Mtb* WT caused a highly significant increase in IL-1β expression (*P* < 0.0001 vs*.* N. Inf.), an effect not observed upon infection with WT ATc (Fig. 8b). Unexpectedly, infection with *Mtb*::PLJR965 reduced IL-1β levels versus WT but did not cause any significant changes compared to non-infected macrophages. IL-1β mRNA levels increased significantly following infection with the induced *Mtb*::PLJR965-sgRNA1-*murT* KDM, compared to N. Inf. ATc (*P* = 0.0146), WT ATc (*P* = 0.0350), and PLJR965 ATc (*P* = 0.0398) (Fig. 8b). A similar tendency was observed following infection with *Mtb*::PLJR965-sgRNA2-*gatD* ATc, although without significance. IL-1β expression was also significantly elevated upon infection with the induced *Mtb*::PLJR965-sgRNA1- *namH* KDM versus N. Inf. ATc (*P* = 0.0004), WT ATc (*P* = 0.0010), PLJR965 ATc (*P* = 0.0011), and the respective uninduced control (*P* = 0.0485) (Fig. 8b).

Infection with WT caused a highly significant rise in IL-10 levels relative to N. Inf. (*P* = 0.0004) (Fig. 8c). Moreover, infection with the induced *Mtb*::PLJR965-sgRNA1-*murT* KDM caused a highly significant downregulation of IL-10 versus WT ATc (*P* = 0.0011), PLJR965 ATc (*P* = 0.0009), and the respective uninduced control (*P* < 0.0001) (Fig. 8c). A similar tendency was observed following infection with *Mtb*::PLJR965-sgRNA2-*gatD* ATc, yet without statistical significance. Conversely, infection with *Mtb*::PLJR965-sgRNA1-*namH* ATc significantly upregulated IL-10 levels compared to N. Inf. ATc (*P* = 0.0025) and the uninduced control (*P* = 0.0122) (Fig. 8c).

## Discussion

Studying PG biosynthesis enzymes and inhibitors offers a promising approach to uncover novel antimycobacterial targets. Alongside increasing clinical reports demonstrating the benefits of β-lactam/β-lactam inhibitor (BL/BLI) combinations against MDR-TB^49–53^, the WHO has included MEM and imipenem-cilastatin in group C of drugs advised for extended MDR-TB regimens^54^. Here, we investigated the roles of D-*i*Glu amidation and muramic acid *N*-glycolylation in *Mtb* growth, β-lactam susceptibility, and host immunity. Our findings revealed that MurT/GatD and NamH contribute to β-lactam resistance, with MurT/GatD also playing a role in *Mtb* intracellular fitness and immune modulation.

The qRT-PCR results demonstrated effective silencing of all GOIs with at least one of the employed sgRNAs. Contrasting previous observations in *Msm*^8,39^ but consistent with findings in *Mtb*^35,36,55^, knockdown efficiency did not correlate with the target site’s distance from the transcription start site. Interestingly, *gatD* repression was more efficient with sgRNA2-*gatD*, despite employing a weaker PAM and targeting a distal ORF region, suggesting that other sgRNA design parameters like GC content and sgRNA length may justify this observation^55^. Collectively, our results suggest that dCas_Sth1_-mediated knockdown produces effective target inhibition in *Mtb*, directly impacting the associated phenotypes.

Phenotyping assays confirmed the essentiality of D-*i*Glu amidation for survival, supporting previous observations in *Msm*^8^ and *Mtb*^7,18,19,48^. The conserved regions producing the Mur_Ligase_M domain of MurT_TB_, an ATP/Mg^2+^ binding site, are targeted by sgRNA2-*murT*^7,11^, likely reducing protein synthesis, enzymatic activity, and ultimately causing the lethal phenotype of the corresponding KDM. Contrarily, sgRNA1-*murT* targets the four conserved amino acid regions (I-IV) within the DUF1727 domain of MurT_TB_, which govern its interplay with GatD and contain catalytic D337^7,8,11^. Nevertheless, since sgRNA1-*murT* targets the 3’-end of *murT* and the employed PAM (PAM9) is relatively weak, RNA polymerase may transcribe most of *murT*, generating a semi-functional protein and facilitating the growth of the respective KDM^8^. Conversely, the GATase_3 domain of GatD_TB_ encompasses conserved residues for glutamine capture (R124) and the channeling of ammonia to MurT_TB_ (G56), along with catalytic residues C90 and H190^7,8,11^. Repression of *gatD* with sgRNAs 1 and 2, which target sites near some of these residues, may lead to depleted D-*i*Glu amidation and reduced PG cross-linking. Since only sgRNA2-mediated repression caused significant *gatD* knockdown, the corresponding KDM displays impaired growth.

In contrast, *namH* is generally classified as non-essential for the growth of *Msm*^8^ and *Mtb*^18,19^. Nonetheless, sgRNA2-mediated *namH* repression resulted in discernible growth deficits, suggesting that *namH* may be conditionally essential, in line with previous evidence of essentiality in *Mtb*^48^. NamH comprises a N-terminal UlaG domain of the metallo-β-lactamase fold, and a C-terminal Rieske domain, which supports muramic acid *N*-glycolylation^8,17^. The sgRNAs employed here target the 5’-end of *namH*. Even though sgRNA1-*namH* targets the promoter zone, the stronger growth inhibition induced by sgRNA2-*namH* may stem from differences in PAM strength and/or specific target site properties.

Furthermore, susceptibility assays demonstrated that both D-*i*Glu amidation and the *N*-glycolylation of muramic acid contribute to β-lactam resistance. In fact, NamH and MurT/GatD have been reported as modulators of β-lactam resistance in mycobacteria^8,9,17^ and *S. aureus*^10,11^. Specifically, sgRNA2-mediated *murT* silencing caused the greatest susceptibility differences relative to WT, suggesting that MurT activity promotes β-lactam resistance. Overall, susceptibility gains were often correlated with growth defects in phenotyping assays, namely in the *murT* and *namH* KDMs.

Additionally, CTX is typically more effective against *Mtb* than AMX, since it inhibits both PBPs and Ldts^27,56^. Curiously, AMX + CLA produced a greater MIC reduction than CTX + CLA, likely because CLA enhances the activity of AMX, while CTX alone is more stable against BlaC-mediated hydrolysis. The greater MIC reduction observed with CLA plus AMX (~ 64-fold) versus CTX or MEM (~ fourfold) may derive from differences in the intrinsic stability of β-lactams against BlaC. Indeed, BlaC has been suggested to preferentially hydrolyse penicillins over cephalosporins or carbapenems^28,57^. The lower MICs observed for MEM versus AMX and CTX are also consistent with its greater stability against BlaC-mediated hydrolysis and its inhibition of both PBPs and Ldts^25,27^. Interestingly, the increased susceptibility of *murT* KDMs to INH, also observed in *Msm*^8^, suggests that MurT activity promotes CW integrity.

Adding to this, disk diffusion assays demonstrated increased β-lactam susceptibility following CRISPRi-mediated inhibition of characteristic mPG modifications. Unlike MurT, GatD depletion significantly increased susceptibility to AMX + CLA and MEM + CLA. Notably, NamH depletion caused β-lactam hypersusceptibility, underscoring the role of muramic acid *N*-glycolylation in the intrinsic β-lactam resistance of WT mycobacteria. Therefore, antibiotics targeting these mPG modifications could synergize with β-lactams to improve TB treatment.

After, checkerboard assays demonstrated that inhibition of characteristic mPG modifications produced reduced FICI_min_ values between EMB and AMX/MEM + CLA. Consistent with previous findings^41^, synergistic effects were more commonly observed with EMB plus AMX + CLA versus MEM + CLA. This likely stems from the substantial enhancement of AMX efficacy when combined with CLA, which is remarkably superior to that observed with MEM + CLA. Also, the FICI_min_ values obtained for the EMB and MEM + CLA combination in WT did not meet the conventional synergy threshold (FICI ≤ 0.5), rather indicating an additive effect, although these were roughly identical to previously reported values^41^. Recently, several studies have reported synergies between penicillins, carbapenems, and cephalosporins with rifampicin and EMB in vitro and intracellularly^52,58,59^. Remarkably, orally administered cephradine and faropenem exhibit synergism with novel anti-TB agents bedaquiline, delamanid, and pretomanid^52^. Here, CRISPRi-mediated NamH depletion, coupled with EMB-induced AG biosynthesis inhibition, and β-lactam-mediated transpeptidase inhibition, resulted in enhanced synergy, consistent with compounded perturbations in CW integrity. Taken together, these findings support the hypothesis that NamH inhibition could synergize with therapeutic schemes containing EMB and AMX/MEM + CLA.

Investigating how these mPG modifications modulate host–pathogen interactions could guide the development of therapeutics that strengthen immune responses against TB. Host pattern recognition receptors (PRRs)-mediated recognition of mPG, a conserved pathogen-associated molecular pattern (PAMP), induces pro-inflammatory responses (e.g., elevated TNF-α and IL-1β levels) against *Mtb*. Soluble PG fragments containing D-glutamyl-*meso*-diaminopimelic acid and muramyl dipeptide (MDP) are recognized by intracellular PRRs NOD1 and NOD2, respectively^60,61^.

In line with this, infection assays showed that MurT/GatD depletion reduced *Mtb* intracellular fitness, consistent with increased macrophage activation and immune cell recruitment, potentially linked to the unmasking of NOD1 ligands^62^, alluding to a role for D-*i*Glu amidation in intracellular fitness. These findings reflect previous in vitro studies in *Msm*^8^ and in vivo studies in *M. bovis* BCG^16^. Supporting former studies^22^, NamH depletion did not significantly affect intracellular fitness, despite its reported contribution to *Msm* survival within host macrophages^8^. This suggests a primary role for NamH in CW integrity rather than intracellular fitness under these conditions.

Moreover, depletion of D-*i*Glu amidation upregulated IL-1β mRNA levels and downregulated IL-10 expression, while inhibition of muramic acid *N*-glycolylation induced both IL-1β and IL-10 expression.

Non-infected ATc-treated THP-1-derived macrophages displayed half the TNF-α expression levels of their untreated counterparts, suggesting that ATc may somehow modulate TNF-α expression in non-infected cells. NamH depletion, which results in a higher proportion of *N*-acetyl-MDP, increased TNF-α expression versus non-infected ATc-treated macrophages, though not significantly compared to the uninduced control, suggesting a non-specific effect. Although *N*-glycolyl-MDP has been suggested to stimulate NOD2-mediated TNF-α production^21,22,63^, the lack of NamH has not been associated with significant differences in *Mtb* intracellular survival^22^ or in human NOD1/2-mediated recognition^15^. Discrepancies between studies using mouse- and human-derived macrophages might be attributed to species-specific basal gene expression and NOD sensor responsiveness. Overall, NamH depletion did not lead to statistically significant differences in cytokine expression, as robust TNF-α expression is mostly elicited when NOD2 is co-activated with other PRRs^64^.

On top of that, infection with *Mtb* WT significantly increased IL-1β expression versus non-infected controls, whereas infection with WT ATc did not alter IL-1β levels. Despite having little effect on *Mtb* growth^65^, ATc may exert immunomodulatory effects^66^. Similarly, infection with *Mtb*::PLJR965 did not affect IL-1β expression, perhaps owing to altered PAMP secretion or stress responses that dampen pro-inflammatory signaling. Notably, MurT/GatD depletion upregulated IL-1β expression, which may be consistent with increased accessibility of NOD ligands and heightened inflammatory signaling. Supporting this, MurT/GatD depletion in *M. bovis BCG* resulted in increased NOD1/2-mediated immune recognition^16^. Furthermore, NamH depletion upregulated IL-1β levels, possibly reflecting increased inflammatory responses associated with altered PG accessibility.

Despite both being pro-inflammatory cytokines, TNF-α and IL-1β mRNA levels differed, likely due to distinct sensitivities, as IL-1β expression, unlike TNF-α, is readily induced through NF-κB signalling^67^.

In this context, IL-10 regulation during TB infection is crucial to prevent excessive inflammation, tissue destruction, and persistence^68,69^. MurT/GatD depletion downregulated IL-10 levels, suggesting a potential role for D-*i*Glu amidation in modulating immune responses linked to persistence. This is aligned with previous reports demonstrating that D-*i*Glu amidation mediates NOD1/2-mediated immune evasion^14,15^. In contrast, NamH depletion upregulated IL-10 expression, possibly as a compensatory response to the concurrent upregulation of IL-1β, whereas muramic acid *N*-glycolylation did not impact IL-10 expression.

Overall, the *N*-glycolylation of mPG does not significantly impact *Mtb* intracellular fitness, while D-*i*Glu amidation may support persistence through immune modulation, consistent with reported roles in immune evasion^14–16^ and anti-inflammatory cytokine expression changes.

Our work has numerous limitations. CRISPRi is typically associated with downstream polarity in operonic genes, in line with the observed reduction in *Rv3819* mRNA levels following *namH* repression^38,39^. Nevertheless, this issue is minimized since *Rv3818*-*Rv3819* are likely functionally related^35,55^. Despite supporting the development of small molecules targeting MurT/GatD and NamH, we recognize that their ability to fully suppress enzyme functions may be limited compared to CRISPRi^55^. In addition, no biochemical or ultrastructural PG analyses were performed, which limits mechanistic interpretations. Besides, immune response differences were assessed by qRT-PCR instead of ELISA, which would have enabled direct cytokine quantification and facilitated comparison with previous studies. Furthermore, cytokine expression was not normalized to intracellular bacterial burden, thus preventing distinction between changes caused by differential PG-mediated immune recognition or secondary effects related to bacterial load.

To conclude, both PG modifications contribute to β-lactam resistance. While NamH is a key resistance modulator, MurT/GatD activity facilitates *Mtb* intracellular fitness and modulates host immune interactions, possibly by playing a role in immune evasion. Our findings suggest that targeting MurT/GatD and NamH, individually or simultaneously, could represent a novel TB-fighting strategy, with the bonus of improving β-lactam efficacy in MDR-TB therapy.

## Supplementary Information

Below is the link to the electronic supplementary material.

Supplementary Material 1

## Data Availability

All data supporting the findings of this study are included in the article and supplementary material. In fact, no novel nucleic acid sequences were generated in this study as Sanger sequencing was only performed to confirm the integrity of sgRNA inserts in a previously described plasmid backbone (PLJR965). In this way, the employed sgRNA sequences and Sanger sequencing results are also included in the Supplementary Information file. Additional information is available from the corresponding author upon request.
