# Supplementary material for: Genetic interference of distinctive Mycobacterium tuberculosis peptidoglycan modifications enhances β-lactam susceptibility and reveals expression-sensitive host immune dynamics
Source: Sci Rep. 2026 Jun 23;16:28660. doi: 10.1038/s41598-026-59172-9 (PMC13574776; doi:10.1038/s41598-026-59172-9)
Supplement: Supplementary file 1 [file 41598_2026_59172_MOESM1_ESM.pdf]

Supplementary Material

1. Supplementary Figures and Tables

1.1. Supplementary Figures

***Mtb*::PLJR965-sgRNA1 *murT* – Sequencing output**

5' – ...ATCAGTGATAGATATAATCTGGGAGCCGGCCGGGTTTTTGGCCAGTTTTTGTACTCGAAAGAAGCTACAAAGATAAGGCTTCATGCCGTAATC... - 3'

***Mtb*::PLJR965-sgRNA2 *murT* – Sequencing output**

5' - ...ATCAGTGATAGATATAATCTGGGAGTCCAGCTGGTCTCGGGAGAGGTGTTTTTGTACTCGAAAGAAGCTACAAAGATAAGGCTTCATGCCGAAATC... – 3'

***Mtb*::PLJR965-sgRNA1 *gatD* – Sequencing output**

5' – ...ATCAGTGATAGATATAATCTGGGAATCTCGGCGGCGATGCCGCGCAGTTTTTGTACTCGAAAGAAGCTACAAAGATAAGGCTTCATGCCGAAATC... – 3'

***Mtb*::PLJR965-sgRNA2 *gatD* – Sequencing output**

5' - ... ATCAGTGATAGATATAATCTGGGAGCCCAAGGGCGACGTTCCGGGCCGTTTTTGTACTCGAAAGAAGCTACAAAGATAAGGCTTCATGCCGAAATC... - 3'

***Mtb*::PLJR965-sgRNA1 *namH* – Sequencing output**

5' – ...CCTGGGGATTTGCGATCCCTATCAGTGATAGATATAATCTGGGAGTGACCTGCACAGCTACCTTGTTTTTGTACTCGAAAGAAGCTACAAAGATAAGGCTTCATGCCGAAATCAACACCCT... – 3'

***Mtb*:: PLJR965-sgRNA2 *namH* – Sequencing output**

5' – ...TGGGGATTTGCGATCCCTATCAGTGATAGATATAATCTGGGAGCTGCCGGCCTGGGTCTGGAGTTTTTGTACTCGAAAGAAGCTACAAAGATAAGGCTTCATGCCGAAAT... – 3'

Supplementary Fig. S1 - Sequencing results (5' – 3') of PLJR965 with the sgRNA targeting each gene of interest (*murT, gatD*, or *namH*) in *M. tuberculosis*. The -10 site is underlined in yellow, the insert or sgRNA is underlined in green and the dCas9 handle is painted in grey.

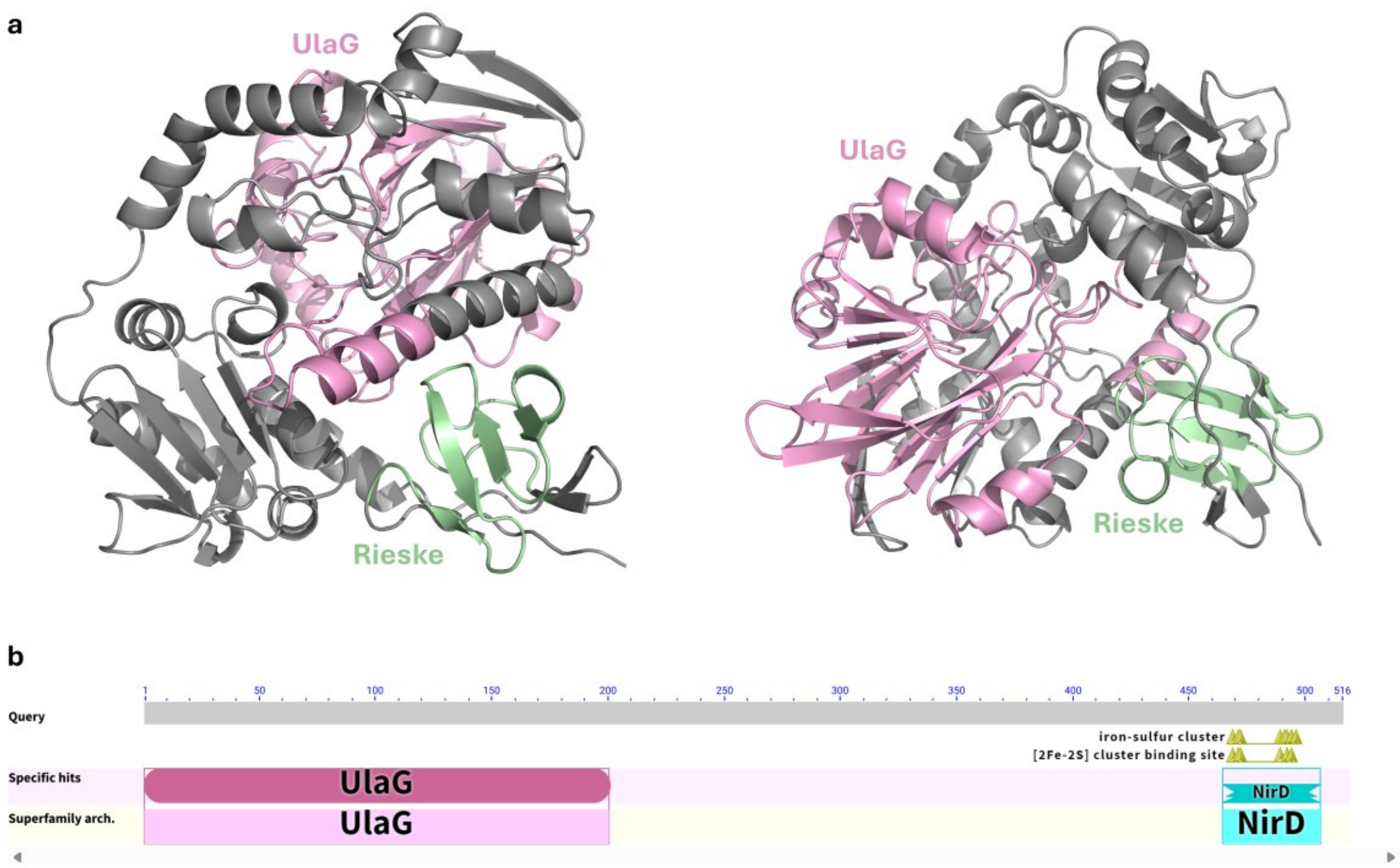

Name	Accession	Description	Interval	E-value
+ UlaG	COG2220	L-ascorbate lactonase UlaG, metallo-beta-lactamase superfamily [Carbohydrate transport and metabolism]	1-200	1.33×10^{-17}
+ NirD Rieske	COG2146	Ferredoxin subunit of nitrite reductase or a ring-hydroxylating dioxygenase [Inorganic ion transport and metabolism, Secondary metabolites biosynthesis, transport and catabolism]	465-506	4.98×10^{-10}

Supplementary Fig. S2 - Domain architecture and structural organization of NamH. (**a**) AlphaFold-predicted three-dimensional structure of NamH shown in two distinct perspectives. The N-terminal UlaG domain (pink), belonging to the metallo-β-lactamase fold, forms the catalytic core. The C-terminal Rieske-type domain (green), corresponding to a NirD-like [2Fe–2S] motif, is likely involved in electron transfer, which supports the *N*-glycolylation of muramic acid. The remaining regions are shown in grey. (**b**) NCBI-predicted domain architecture of NamH, based on conserved domain and COG analysis. The protein is annotated as "an uncharacterized MBL fold metallo-hydrolase containing a Rieske (2Fe-2S) domain".

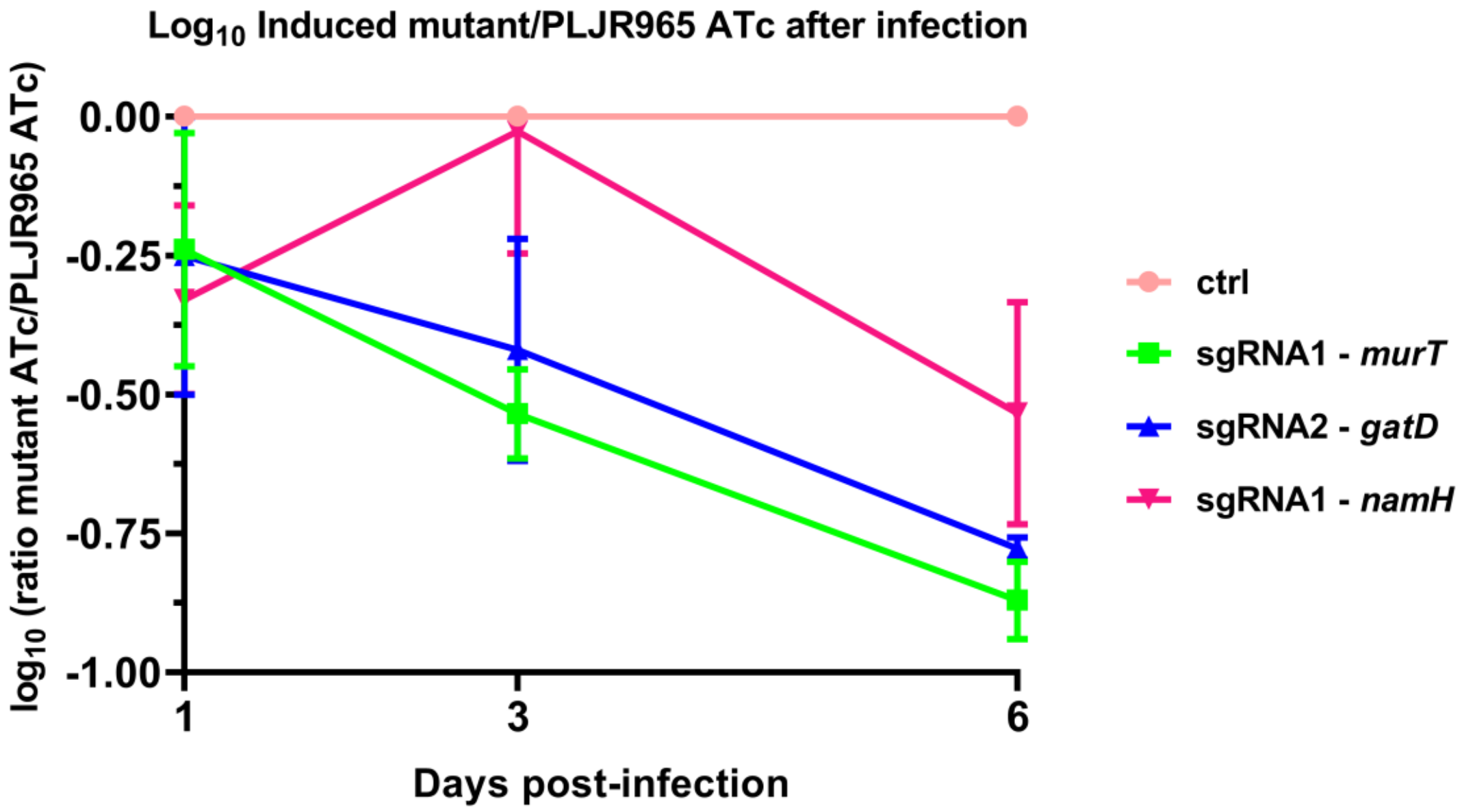

Supplementary Fig. S3 – Mean $\log_{10}$ ratios of CFU/mL for the induced *murT* (sgRNA1), *gatD* (sgRNA2) and *namH* (sgRNA1) knockdown mutants relative to the induced control strain *Mtb* PLJR965 (ctrl) (at 1, 3, and 6 days post-infection. Ratios were calculated as CFU/mL (mutant+ATc) divided by CFU/mL (PLJR965+ATc). Values below 0 indicate reduced intracellular growth relative to the induced control vector (PLJR965+ATc). Error bars represent the standard error of the mean (SEM) of the log-transformed values. This supplemental figure provides a normalized visualization of the intracellular survival trends corresponding to those shown in Fig. 7. No statistical testing was performed; data are presented for descriptive purposes only.

1.2. Supplementary Tables

Supplementary Table S1 - Bacterial strains, plasmids, and cell lines used in this study.

Bacterial strain, plasmid or cell line	Description	Reference or source
Bacteria		
Escherichia coli		
JM109	*recA1 endA1 gyr96 thi hsdR17 supE44 relA1* Δ(*lac-proAB*) [F´ *traD36 proAB lac*^{q}ZΔM15]	Stratagene
Mycobacterium tuberculosis		
H37Rv	Virulent strain derived from *M. tuberculosis* H37	25618™, ATCC
Plasmids		
PLJR965 (8631 bp)	Sth1 *dcas9* Tet^{R} and Kan^{R} L5 Int *attP* for *M. tuberculosis*	35
Cell lines		
THP-1	Human-derived monocyte cell line	TIB-202™, ATCC

Supplementary Table S2 – sgRNAs used to target *murT*, *gatD*, and *namH* in *M. tuberculosis*. [a] - PAM strength was assessed in accordance with the table repertoire of functional PAMs *in vivo* for $dCas9_{Sth1}$ defined by Rock et al., 2017 [35]; [b] - mRNA knockdown caused by the electroporation of the indicated sgRNA in the *M. tuberculosis* WT strain was measured by qRT-PCR after ATc induction. Fold knockdown efficiency is relative to an uninduced control plasmid containing the same sgRNA.

Target gene	Name of the oligos	PAM	PAM strength[a]	Gene Location 5'-3' (bp)	Base-pairing region of the sgRNA sequence (the 12 bp seed region is underlined)	BLASTed sequence in the genome of *Mtb* (seed region + PAM)	Fold knockdown efficiency[b]
murT (*Rv3712*)	sgRNA1	5' - GCAGGAT - 3'	PAM9	927	5' - GCCGGCCGGGTTTTTGGCCA - 3'	5' - GGTTTTTGGCCAGCAGGAT - 3'	10.1
	sgRNA2	5' - TGAGCAA - 3'	PAM10	411	5' - GTCCAGCTGGTCTCGGGAGAGGT - 3'	5' - CTCGGGAGAGGTTGAGCAA - 3'	5.7
gatD (*Rv3713*)	sgRNA1	5' - GCAGCAG - 3'	PAM13	110	5' - ATCTCGGCGGCGATGCCGCGCA - 3'	5' - CGATGCCGCGCAGCAGCAG - 3'	1.9
	sgRNA2	5' - CGAGGAC - 3'	PAM15	486	5' - GCCCAAGGGCGACGTTCCGGGCC - 3'	5' - ACGTTCCGGGCCCGAGGAC - 3'	7.0
namH (*Rv3818*)	sgRNA1	5' - TCGGAAG - 3'	PAM4	11	5' - GTGACCTGCACAGCTACCTT - 3'	5' - CACAGCTACCTTTCGGAAG - 3'	8.3
	sgRNA2	5' - TCAGAAA - 3'	PAM3	57	5' - GCTGCCGGCCTGGGTCTGGA - 3'	5' - CCTGGGTCTGGATCAGAAA - 3'	8.3

Supplementary Table S3 - Primers designed and synthesized to clone the targeting sgRNAs into PLJR965 for CRISPRi-mediated targeting in *M. tuberculosis*. [a] – PAM strength was described in accordance with the table of functional PAMs *in vivo* for $dCas9_{Sth1}$-mediated targeting in mycobacteria defined by Rock *et al.*, 2017 [35]; [b] – Calculated with the Eurofins Genomics Melting temperature (T_m) formula.

Target gene	Name of the oligos	PAM	PAM strength[a]	Gene Location 5'-3' (bp)	Primers		Length (bp)	% GC	T_m[b] (° C)
murT (*Rv3712*)	sgRNA1	5' - GCAGGAT - 3'	PAM9	927	PFwd	5' - GGGAGCCGGCCGGGTTTTTGGCCA - 3'	24	71	71
					PRv	5' - AAACTGGCCAAAAACCCGGCCGGC - 3'		63	68
	sgRNA2	5' - TGAGCAA - 3'	PAM10	411	PFwd	5' - GGGAGTCCAGCTGGTCTCGGGAGAGGT - 3'	27	67	73
					PRv	5' - AAACACCTCTCCCGAGACCAGCTGGAC - 3'		59	70
gatD (*Rv3713*)	sgRNA1	5' - GCAGCAG - 3'	PAM13	110	PFwd	5' - GGGAATCTCGGCGGCGATGCCGCGCA - 3'	26	73	74
					PRv	5' - AAACTGCGCGGCATCGCCGCCGAGAT - 3'		65	71
	sgRNA2	5' - CGAGGAC - 3'	PAM15	486	PFwd	5' - GGGAGCCCAAGGGCGACGTTCCGGGCC - 3'	27	78	77
					PRv	5' - AAACGGCCCGGAACGTCGCCCTTGGGC - 3'		70	74
namH (*Rv3818*)	sgRNA1	5' - TCGGAAG - 3'	PAM4	11	PFwd	5' - GGGAGTGACCTGCACAGCTACCTT - 3'	24	58	66
					PRv	5' - AAACAAGGTAGCTGTGCAGGTCAC - 3'		50	63
	sgRNA2	5' - TCAGAAA - 3'	PAM3	57	PFwd	5' - GGGAGCTGCCGGCCTGGGTCTGGA - 3'	24	75	73
					PRv	5' - AAACTCCAGACCCAGGCCGGCAGC - 3'		67	70

Supplementary Table S4 - Primers used to quantify the mRNA expression levels of target genes *murT*, *gatD,* and *namH* by qRT-PCR in *M. tuberculosis*. [a] - qRT-PCR primers were designed to avoid primer secondary structures and sequence repeats, have at least 50% of GC content, and have a T_m between 57-63°C; [b] - Calculated with the Eurofins Genomics T_m formula; [c] – AE designates the amplification efficiency calculated for each pair of primers in a single-run qPCR amplification of the calibrator sample (*M. tuberculosis* WT), performed in triplicates.

**qRT-PCR primers[a]**							
**Target gene**		**Primer sequences**	**Length (bp)**	**% GC**	**T_m[b] (°C)**	**Product size (bp)**	**AE[c] (%)**
***sigA* (*Rv2703*)**	PFwd	5' - CGCGACATGATGTGGATCTG - 3'	20	55	59	186	80
	PRv	5' - CCCCTTGGTGTAGTCGAACT - 3'	20	55	59		
Rv3711c	PFwd	5’ – GCTGCCCTAGAGAGTGC – 3’	17	65	58	95	87
	PRv	5’ – TCGTCGTGAGTCACCCG – 3’	17	65	58		
***murT* (*Rv3712*)**	PFwd	5’ – GGGCGGGTTCCTGACG – 3’	16	75	60	180	104
	PRv	5'- TGGGCATGAGGCGATGG – 3’	17	65	58		
***gatD* (*Rv3713*)**	PFwd	5’ – GGCGACGGTTTTGATGGC – 3’	18	61	58	146	99
	PRv	5’ – TCCACCTCGGGCAAATCC – 3’	18	61	58		
***namH* (*Rv3818*)**	PFwd	5’ – GTGTTCCTGGATCAGATGCG – 3’	20	55	59	149	92
	PRv	5’ – TTGTCGGTGGTAAAGATGGC – 3’	20	50	57		
Rv3819	PFwd	5' - TGGCTGCCACTGGACGTG - 3'	18	67	61	142	85
	PRv	5' - GGTGGAGGGCGAGCAACTC - 3'	20	68	63		

Supplementary Table S5 - Primers used to quantify the mRNA expression levels of target genes *TNF-α*, *IL-1β*, and *IL-10* by qRT-PCR in *Mtb*-infected THP-1-derived macrophages. [a] - qRT-PCR primers were designed to avoid primer secondary structures and sequence repeats, have at least 50% of GC content, and have a T_m between 57-63°C; [b] - Calculated with the Eurofins Genomics T_m formula; [c] – AE designates the amplification efficiency calculated for each pair of primers in a single-run qPCR amplification of the *Mtb* PLJR965-infected THP-1-macrophage sample, performed in triplicates.

qRT-PCR primers[a]								
**Target transcript**		**Primer sequences**	**Length (bp)**	**% GC**	**T_m[b] (°C)**	**Product size (bp)**	**AE[c] (%)**	**Reference**
GAPDH	PFwd	5' - CTGGGCTACACTGAGCACC - 3'	19	63	61	101	86	Jiang & Li, 2022
	PRv	5' - AAGTGGTCGTTGAGGGCAATG - 3'	21	52	60			
TNF-α	PFwd	5' - CCAGGGACCTCTCTCTAATC - 3’	20	55	59	83	112	Shu *et al.*, 2022
	PRv	5' - ATGGGCTACAGGCTTGTCACT - 3’	21	52	60			
IL-1β	PFwd	5' - TACTCACTTAAAGCCCGCCT - 3'	20	50	57	77	94	Medeiros-Furquim *et al.*, 2022
	PRv	5' - ATGTGGGAGCGAATGACAGA - 3'						
IL-10	PFwd	5' - GCTGGAGGACTTTAAGGGTTACCT - 3'	24	50	63	109	117	Demers-Mathieu, Huston & Dallas, 2020
	PRv	5' - CTTGATGTCTGGGTCTTGGTTCT - 3'	23	48	61			

References:

- Jiang, L. & Li, J. lncRNA GMDS-AS1 upregulates IL-6, TNF-α and IL-1β, and induces apoptosis in human monocytic THP-1 cells via miR-96-5p/caspase 2 signaling. *Mol. Med. Rep.* **25**, 1–9; 10.3892/mmr.2022.12583 (2022).
- Demers-Mathieu, V., Huston, R. K. & Dallas, D. C. Cytokine expression by human macrophage-like cells derived from the monocytic cell line THP-1 differs between treatment with milk from preterm- and term-delivering mothers and pasteurized donor milk. *Molecules* **25**, 2376; 10.3390/molecules25102376 (2020).
- Medeiros-Furquim, T. *et al*. Cladribine treatment for MS preserves the differentiative capacity of subsequently generated monocytes, whereas its administration *in vitro* acutely influences monocyte differentiation but not microglial activation. *Front. Immunol.* **13**, 1–16; 10.3389/fimmu.2022.678817 (2022).
- Shu, F., Thammasit, P., Pruksaphon, K., Nosanchuk, J. D. & Youngchim, S. Expression of cytokine profiles in human THP-1 cells during phase transition of *Talaromyces marneffei*. *Pathogens* **11**, 1465; 10.3390/pathogens11121465 (2022).
